# Ambrosin, a potent NF-κβ inhibitor, ameliorates lipopolysaccharide induced memory impairment, comparison to curcumin

**DOI:** 10.1371/journal.pone.0219378

**Published:** 2019-07-05

**Authors:** Mohammed N. A. Khalil, Mouchira A. Choucry, Amira S. El Senousy, Azza Hassan, Salma A. El-Marasy, Sally A. El Awdan, Farghaly A. Omar

**Affiliations:** 1 Pharmacognosy Department, Faculty of Pharmacy, Cairo University, Cairo, Egypt; 2 Pathology Department, Faculty of Veterinary Medicine, Cairo University, Giza Square, Giza, Egypt; 3 Pharmacology Department, National Research Centre, Giza, Egypt; 4 Pharmaceutical Chemistry Department, Faculty of Pharmacy, Assiut University, Assiut, Egypt; Sungkyunkwan University, REPUBLIC OF KOREA

## Abstract

Despite its poor bioavailability, curcumin is a promising natural polyphenol targeting NF-κβ. NF-κβ is a target for new therapeutics because it plays a pivotal role in the pathophysiology of Alzheimer disease (AD). In contrast, ambrsoin, a sesquiterpene lactone which is a potent NF-κβ inhibitor, is scarcely studied in AD models. The current work aims to assess the efficacy of ambrosin as a possible remedy for AD. *In silico* studies showed that bioavailability and BBB permeability could be favorable for ambrosin over curcumin. Memory impairment was induced in mice by single intraperitoneal injection of LPS (0.4 mg/kg). Treated groups received curcumin (100 mg/kg) or ambrosin at doses (5 or 10 mg/kg) for 7 days. Mice in treated groups showed a significant improvement in memory functions during Morris water maze and object recognition tests. Curcumin and ambrosin (10 mg/kg) inhibited the upsurge of NF-κβp65 transcript and protein levels. Consequently, downstream pro-inflammatory and nitrosative mediators were inhibited, namely, TNF-α, IL-1β, COX-2 and iNOS. BACE1 was inhibited, thereby reducing amyloid plaques (Aβ) deposition and eventually reducing inflammation and apoptosis of neurons as revealed by immunohistopathological examination. In conclusion, ambrosin can be repurposed as AD remedy after further pharmacokinetic/pharamacodynamic assessments. It could serve as an additional lead drug for AD therapeutics.

## Introduction

Alzheimer disease (AD) is the most prevalent form of dementia in late ages. The histopathological hallmarks of the disease are the deposition of intracellular neurofibillary tangles (hyperphosphorylated tau protein) and the extracellular senile plaques of amyloid-beta peptides (Aβ plaques). So far, no disease-modifying drug for AD is available. Drugs inhibiting the formation of Aβ plaques, e.g., γ-secretase, BACE1 inhibitors and amyloid vaccines have failed in clinical trials [1]. Consequently, treatment paradigm is shifted from targeting Aβ deposition and clearance to halting the inflammation process which is predisposing to, and associated with the progression of AD [2].

The transcription factor NF-κβ plays a crucial role in Alzheimer pathophysiology. NF-κβ activation results in the upregulation of Aβ producing genes, *viz*., BACE1, pro-inflammatory cytokines and oxidative stress-related genes [3, 4]. Therefore, NF-κβ is one of the novel targets for alleviation of AD. In this aspect, plant polyphenols are considered as novel candidates for treatment of AD [5]. Among these, curcumin is considered as the most promising remedy [6]. However, its poor bioavailability caused poor efficacy in some clinical trials [5]. Modern pharmaceutical technologies were performed to improve its bioavailability [7]. However, from the phytochemical perspectives, several phytochemicals are known for their NF-κβ inhibitory activity [8]. These Phytochemicals could have better pharmacokinetics and/or pharmacodynamic properties than what curcumin has.

**Fig 1:**
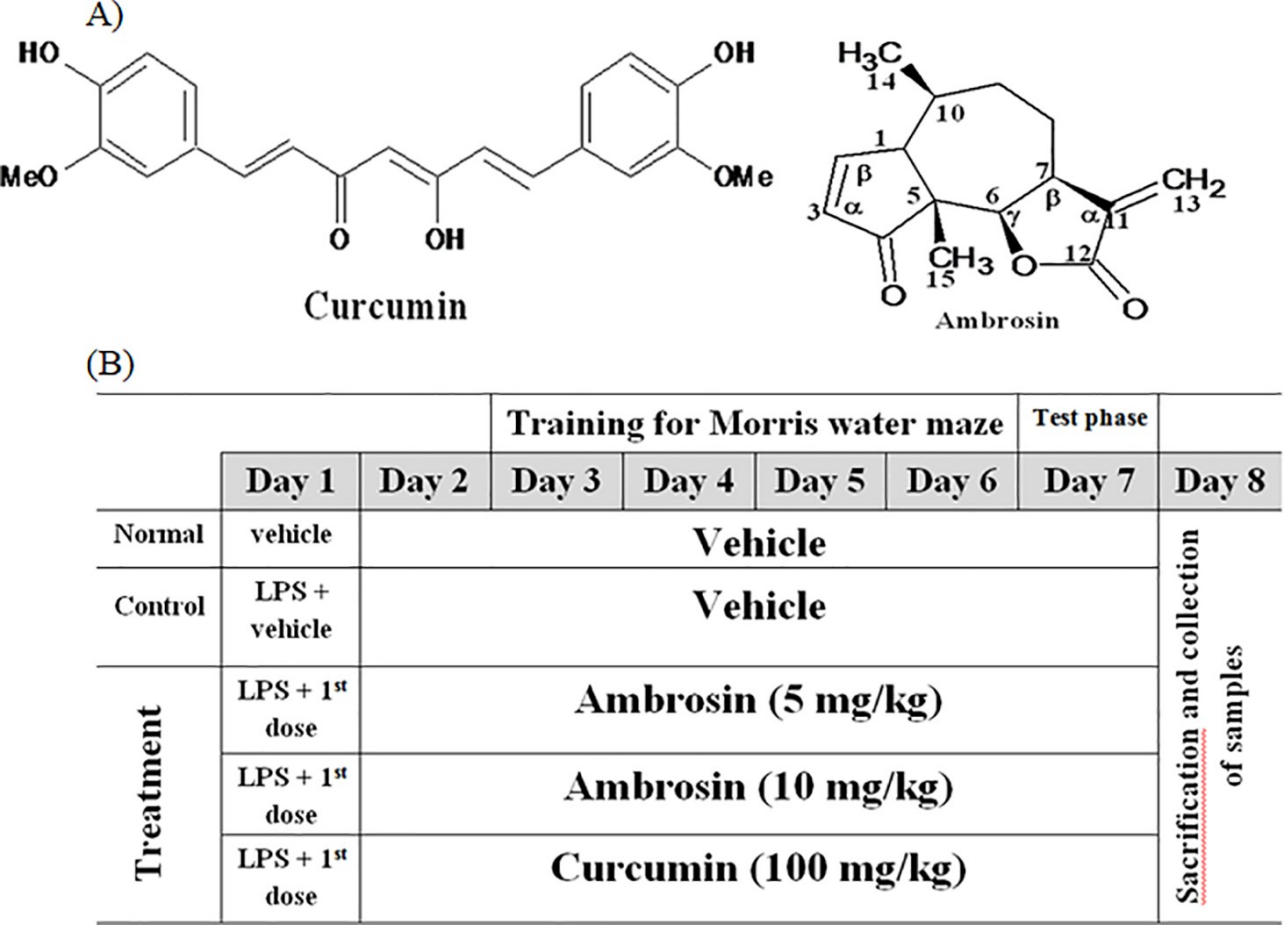
Chemical structures of Curcumin and Ambrosin (A) and diagrammatic table of the experimental design (B).

Ambrosin is a pseudoguainolide sesquiterpene lactone. It is a major phytochemical of many ragweeds species distributed worldwide [9, 10] It has a very potent anticancer activity [11]. Ambrosin was evidenced to be a potent NF-κβ inhibitor in *in vitro* assays [11, 12]. Its chemical scaffold is devoid of any phenolic moiety and it is nonpolar (**Fig 1A)**. Theoretically, it should be as effective as curcumin with a better bioavailability in brain tissues. The aims of the present study were: i) performing *in silico* studies to evaluate the physicochemical and pharmacokinetic properties of ambrosin in comparison to curcumin, and ii) evaluating the therapeutic efficacy of ambrosin in *in vivo* model of AD. Concomitant to the introduction of curcumin as a safe remedy, other phytochemicals of different classes should not be overlooked. The current study addresses the efficacy of one of sesquitepene lactones in alleviating AD and draws attention to this biologically active phytochemical class.

## Materials and methods

Lipopolysaccharides from *Escherichia coli* (O111:B4) was purchased from Sigma-Aldrich (Germany). Silica gel and Sephadex LH-20 for column chromatography, HPLC-grade solvents and TLC plates were purchased from Sigma-Aldrich (Germany). All organic solvents were of analytical grade unless otherwise specified (El-Nasr, Egypt).

### *In silico* prediction of pharmacokinetic properties of ambrosin and curcumin

Pharmacokinetic properties of ambrosin and curcumin were predicted using three online tools, First: ADMET @ LMMD (http://lmmd.ecust.edu.cn/admetsar1/predict/), it is run by Laboratory of Molecular Modeling and Design, Shanghai Key Laboratory of New Drug Design, School of Pharmacy, East China University of Science and Technology [13] second: SwissADME portal (http://www.swissadme.ch/), it is run by Swiss Institute of Bioinformatics [14]. Third: Molinspiration property engine v2016.10 (www.molinspiration.com), it is run by Molinspiration Chemoinformatics.

### Plant material

Aerial parts of *A*. *maritima* were harvested from the Botanical Garden of Faculty of Pharmacy, Cairo University. Identity of the plant was authenticated by Prof. Dr. Wafaa M. Amer, Botany Department, Faculty of Science, Cairo University. A voucher specimen was prepared and deposited in Cairo University herbarium (CAI). Aerial parts were collected excluding large stems and shade-dried, then was grinded coarsely.

### Isolation and structure elucidation of ambrosin

Air-dried aerial parts of *A*. *maritima* (4 kg) were extracted by maceration in 70% ethanol (10 l). The filtrate was evaporated under vacuum at 40°C. Extraction was repeated four times to yield an alcoholic extract (800 g). The alcoholic residue (700 g) was suspended in water and defatted by exhaustive extraction with pet. ether (9 X 250 ml); it yielded 100 g. The defatted extract was shaken with dichloromethane (DCM) (9 x 250 ml) to yield 150 g DCM extract. Portion of the DCM extract (100g) was fractionated using VLC (400g silica, 15 x 8 cm) starting from DCM: pet. ether (50:50) with 10% increments till 100% DCM, then increasing polarity using EtOAc with 10% increment till 100% EtOAc. Fractions were concentrated and monitored by TLC and fractions having similar spots were pooled together. Dragendorff`s reagent and *p*-anisaldehyde were used as spray reagents. Fractions (8–10, 20–40% EtOAc/DCM) contained major sesquiterpene lactones. Ambrosin was isolated by repeated column chromatography where it was eluted with 30–33% EtOAc/pet. ether followed by filtration through sephadex column using mobile phase DCM:MeOH (50:50). Ambrosin (1g) was yielded in the form of needle crystals of 95.1% purity.

### HPLC test for purity

HPLC Agilent 1200 infinity instrument was equipped with manual injector and DAD detector. The mobile phase consisted of solvent A (water containing 0.1% TFA) and solvent B (ACN). The separation was in gradient mode and started by using solvent B at 43% for 2 minutes, then, it was gradually increased to attain 75% at 34 minutes then increased to 100% at 36 minutes. Afterwards the column was washed using 100% MeOH for 2 minutes. Detection wavelengths were set at 225 and 240 nm. The purity of ambrosin was confirmed by HPLC analysis (**S1 Fig).**

### Structure elucidation of ambrosin

Mass scanning and fragmentation were acquired by direct injection in Agilent 6420 triple quadrupole (Agilent, LC-MS QQQ 6420). The MS was run in the positive ion mode with 10 kV as capillary voltage. Source temperature was adjusted at 200°C. Meanwhile, highly pure nitrogen was utilized as auxiliary and sheath gas at flow rates of 40 and 80 arb. unit, respectively. MS/MS fragmentation was conducted at collision energy of 15 ev. In a full scan mode, ions were traced within 50–2000 m/z mass range. NMR was conducted on Bruker High Performance Avance III (400 MHz) NMR spectrometer. One dimensional ^1^H- and ^13^C-NMR as well as two dimensional HMBC and HSQC were performed. NMR data are recorded in **S1 Table** while HMBC and NOESY correlations are illustrated in **S2 Fig**. ESIMS (positive ion mode) m/z (rel. int.): 247.14 [M+H]^+^, (100); 229.23 [M+H-18 (H_2_O)]^+^, (23); 201.05 [M+H-(H_2_O + CO)]^+^, (28); 173.05 [M+H-(H_2_O + 2CO)]^+^, (38); 159 (11); 92.82 (14). Data comply with the previously published literature [9, 15].

### LPS induced memory deterioration in mice

#### Animals

Mature male albino mice, 120–130 g, were provided by the National Research Centre Animal House (Dokki, Giza, Egypt). They were housed in standard polypropylene cages where standard environmental conditions were maintained with equal light-dark cycles. Before the inception of the experiment, animals were adapted for 1 week. They were provided with normal pellet diet of mice and water ad libitum as the same common procedure.

#### Ethics statement

This experimental procedure were conducted by strictly following the guidelines in the Guide for the Care and Use of Laboratory Animals which was published by the US National Institute of Health (NIH Publication No. 85–23, revised 1996) and following the regulations of Animal Care and Use of National Research Centre in Egypt. The experimental protocol was approved by Research Ethics Committee of Faculty of Pharmacy, Cairo University (Approval No.: MP 2181). All procedures were made to minify the animals suffering.

#### Experimental design

Brain neuroinflammation and amyloidogenesis were performed as previously indicated [16]. Induction was incited by a single i.p. dose of LPS (0.4 mg/kg, in 1% Tween 80 / normal saline); higher doses were lethal to mice. Ambrosin and curcumin were prepared in the same vehicle. Mice were randomly allocated to five groups (8 animals each) **(Fig 1B)**. Group I: (normal group) which received daily injection of vehicle only. Group II (control group, Alzheimer non-treated group) received single injection of LPS and then daily injection of vehicle. Groups III & IV (ambrosin-treated groups) after LPS injection, they received ambrosin at dose levels, 5 and 10 mg/kg/day (i.p.) respectively. Group V (curcumin-treated group), after LPS injection, they were administered curcumin at dose level 100 mg/kg/day (i.p.). The treatments for all groups lasted for 7 days. The first doses of treatments or vehicle were administered one hour after LPS injection. The test phases of the behavioral studies were conducted one hour after the last dose administration. All members of each group (8 mice) have performed the behavioral tests. Twenty four hours later, mice were sacrificed by decapitation. After sacrification and brain removal, randomly two brains from each group were reserved for histopathological investigation. The remaining brains (n = 6) were individually homogenized and utilized for the biochemical and molecular investigations. Brains were removed then rinsed with ice-cold isotonic saline solution. Tissue samples were homogenized in phosphate buffer (0.1 M, pH 7.4, ice cold) at ratio 1:10 times (w/v). Supernatant was separated after centrifugation (10.000 x g, 15 min), then aliquots were utilized for biochemical measurements. Ambrosin was not tested previously *in vivo*. However, the usual dose of its analogue parthenolide is 5 mg/kg/day i.p. [17]. Therefore, ambrosin was examined at two dose levels, namely, 5 & 10 mg/kg. Curcumin was examined at 100 mg/kg [18].

#### Morris water maze (MWM)

Assessments of spatial learning and memory were examined using Morris water maze (MWM) as reported by **Bromley-Brits, Deng [19].** The maze was made of a large circular pool (diameter X water depth; 150 X 50 cm). Temperature was maintained at room temperature. A platform (10 cm in diameter) was fixed in the pool.

MWM test was carried out for five consecutive days prior decapitation. On day 1, mice were allowed to carry on a visible platform training session, where each mouse received 5 trials. The position of platform and the starting point (water entry point) for each mouse was changed in every trial. Each mouse was trained to reach the platform in 60 s, the escape latency is recorded as the time required reaching the platform in 60 sec.

On days 2–4, hidden platform training session was carried out. The location of platform was fixed and maintained at height 1 cm below the water surface. The water entry point for each mouse differed in each trial. The mean escape latency time for the daily training sessions was recorded.

On day 5, probe session was carried out, where the platform was removed and the mice were placed at a fixed water entry point. The time spent in the target quadrant (the quadrant where the platform was retained in the training sessions) was calculated **[20]**.

#### Objectr test (ORT)

Object recognition test (ORT) was constructed as described by Mazumder, Sharma [21]. ORT was designed as a black wooden box (40X40X30 cm) opened from the top. The test was carried out for three successive days before sacrifice. On the first day, habituation phase was performed where mice were left to explore the apparatus for 5 min. On the second day, a familiarization or training phase was performed, where two identical objects were laid in the box, and mice were left to explore the objects for 3 min. 24 h later, test phase was performed, where novel object (N) was placed instead of one of the objects, then mice were let to explore the 2 different objects; the novel one (N) and the familiar (F) for 3 min.

Exploration was counted when the mouse touched the object using his nose or directed his nose toward the object at a distance ≤ 2 cm. Based on this, series of variables were determined; the time consumed in exploring the two different objects, N & F was calculated. The discrimination between F and N was measured by comparing the exploration time of F with that of N. DI (discrimination index) expressed the proportion of the exploration times of the two objects. DI was then calculated; DI = N-F/N, similar to previously published [22].

#### Assessment of pro-inflammatory biomarkers

Total NF-κβp65 protein was determined using Invitrogen ELISA Kit; it is a solid phase sandwich ELISA kit type (Invitrogen, CA, USA).

Total RNA was isolated from brain homogenate using QIAamp RNA Mini kit (Qiagen, Germany). Transcript levels of mRNA encoding NF-κβp65 were determined using NF-κβp65 PCR fluorescence quantitative kit cat. No. BAS09S6 which utilizes sybergreen technology (SNP biotechnology, USA). Quantification was performed using Rotor-Gene Q5 plex real-time Rotary analyzers.

TNF-α, COX-2 protein levels were determined using the corresponding ELISA kit (Cusabio, China). iNOS protein levels were estimated using ELISA Kit (EIAab, Germany). IL-1β was estimated using the specific ELISA Kit (Cohesion Biosciences Ltd, UK).

#### Assessment of BACE1 levels

BACEI protein levels were assessed using ELISA Kit (Elabscience, USA). Procedure was as stated by manufacturer.

#### Histopathological examination

The brains were fixed in 10% neutral buffer formalin for at least 48 hours. Then the brain tissues were washed, dehydrated in alcohol and embedded in paraffin blocks. Tissue sections, 4μm thickness, were stained with H&E for preliminary histopathological examination. Additionally, Congo red stain was applied to demonstrate amyloid plaques. To assess the neuronal loss, the surviving neurons in the hippocampal CA1 region were quantified in three high microscopic power field (40X) according to the method of [23]. Ten random microscopic fields (20X) were used in quantification of amyloid plaques, according to the method of [20].

#### Immunohistochemical analysis

Immnuohistochemical staining was carried out to demonstrate neuroinflammation markers, such as COX-2 and CD68, and apoptotic marker as cleaved Caspase-3. Brain sections, paraffin-embedded, were dewaxed and incubated in hydrogen peroxide (3%) to block endogenous peroxidase. After washing in PBS, the sections were incubated with polyclonal anti-COX-2 (Santa Cruz Biotechnology, Santa Cruz, CA; 1:100 dilution, Cat#:sc-1745), mouse monoclonal anti-CD68 (ED1, Abcam, Ltd., USA, 1:1000 dilution, Cat#:ab31630) and rabbit polyclonal anti-caspase-3 (Abcam, Ltd., USA, 1:1000 dilution, Cat#:ab49822) as primary antibodies. Immune reaction was visualized with 3, 4-diaminobenzidine (DAB). Optical density (OD) measurement of COX2, CD68 and cleaved caspase-3 staining in the cerebral cortex of control and treated mice was determined in ten random high power fields.

#### Statistical analysis

Results were expressed as mean ± standard error. Data were analyzed using one way ANOVA followed by Tukey post hoc for multi-comparison test except for object recognition test (ORT) where Student′s t-test was used to test significance of exploration time. Statistical analyses were conducted utilizing the software GraphPad Prism (version 5.0). The differences were counted as significant at *p* < 0.05.

### Molecular docking analysis of ambrosin and curcumin to NF-κβp65

Molecular docking study was performed in the CADD Lab. (Dept. of pharmaceutical Chemistry; Faculty of Pharmacy; Assiut University) utilizing the software Molecular Operating Environment ‎‎(MOE) version 2018.09 (Chemical Computing Group Inc., Montreal, Canada). The software was run under “Windows XP” installed on a PC; its specifications were Intel Pentium IV, processor of 1.6 GHz and memory of 512 MB.

The X-ray crystallographic structure of NF-ҡβp65 dimer linked to DNA (PDB ID: ‎‎1VKX) was extracted from the Protein Data ‎Bank (http://www.rcsb.org/pdb) and prepared for docking studies by adding hydrogen atoms to the system with their standard geometry. The atoms connections and types were checked for any errors using automatic correction. MOE Alpha Site Finder was utilized for the active site search in the protein structure using all default items. The builder interface of the MOE program was used to construct both curcumin and ambrosin into a 3D model. Their structures were checked and subjected to conformational search where all conformers were submitted to energy optimization. All the minimizations were underwent with MOE until a RMSD gradient of 0.01 Kcal/mole and RMS distance of 0.1 Å with MMFF94X force-field and the partial charges were calculated automatically. The obtained database files were then saved as MDB (Molecular Data Base) file to be used in the docking calculations. Docking of the conformers’ database was done using MOE-Dock software. The targets active site file was loaded and the Dock tool was initiated. The program specifications were adjusted to: i) ligand atoms as the docking site, ii) triangle matcher as the placement methodology to be used and iii) London dG as Scoring methodology to be used and was adjusted to its default values.

## Results

An approach was followed to test the potential of ambrosin as a phytochemical candidate, similar to curcumin, for AD therapy. Initially, its pharmacokinetics properties were determined *in silico*, and then it was chemically isolated and purified. Finally, its efficacy was assessed in LPS-induced memory impairment mouse model. Molecular docking was performed to give insights about possible binding sites to active site of NF-κβp65.

### *In silico* prediction of pharmacokinetic properties

Different tools were used to compare the pharmacokinetic properties of curcumin and ambrosin, the results are presented in **Table 1**. The molinspiration platform showed that ambrosin had lower LogP value, half that of curcumin, which indicated better lipid solubility and hence enhanced absorption. Moreover, ambrosin had a lower TPSA (Total molecular Polar Surface Area) which was a further confirmation of its lower polarity and hence better oral bioavailability [24]. SwissADME model predicted that ambrosin could cross BBB but curcumin could not. Similarly, ambrosin had higher probability to cross BBB as predicted by the tool from ADMT @ LMMD portal. However, the last two models predict that both ambrosin and curcumin would be absorbed from GIT. Permeability across Caco-2 cells is indicative about the possibility of drug absorption through intestinal wall [25]. Collectively, these data confirmed the speculations that ambrosin could have a better pharmacokinetic properties regarding reduced polarity and enhanced BBB permeability. These promising properties were incentive to isolate ambrosin and test its therapeutic efficacy in an *in vivo* model.

**Table 1:** Comparison of *in silico* predictions of pharmacokinetic properties of ambrosin and curcumin using different tools.

Tool	Criteria	Ambrosin	Curcumin
Molinspiration	miLogP	**1.26**	**2.3**
	TPSA (Total molecular Polar Surface Area)	**43.37**	**93.06**
SwissADME	GIT Absorption	**High**	**High**
	BBB permeability	**Yes**	**No**
admetSAR@ LMMD	BBB permeability	**0.95**	**0.62**
	Human intestinal absorption	**0.99**	**0.95**
	Caco-2 permeability	**0.62**	**0.71**
	Predicted Caco-2 permeability	**1.33**	**0.65**

### Behavioral tests

#### Curcumin and ambrosin ameliorated learning and spatial memory dysfunctions induced by LPS injection, performance in MWM

**Fig 2:**
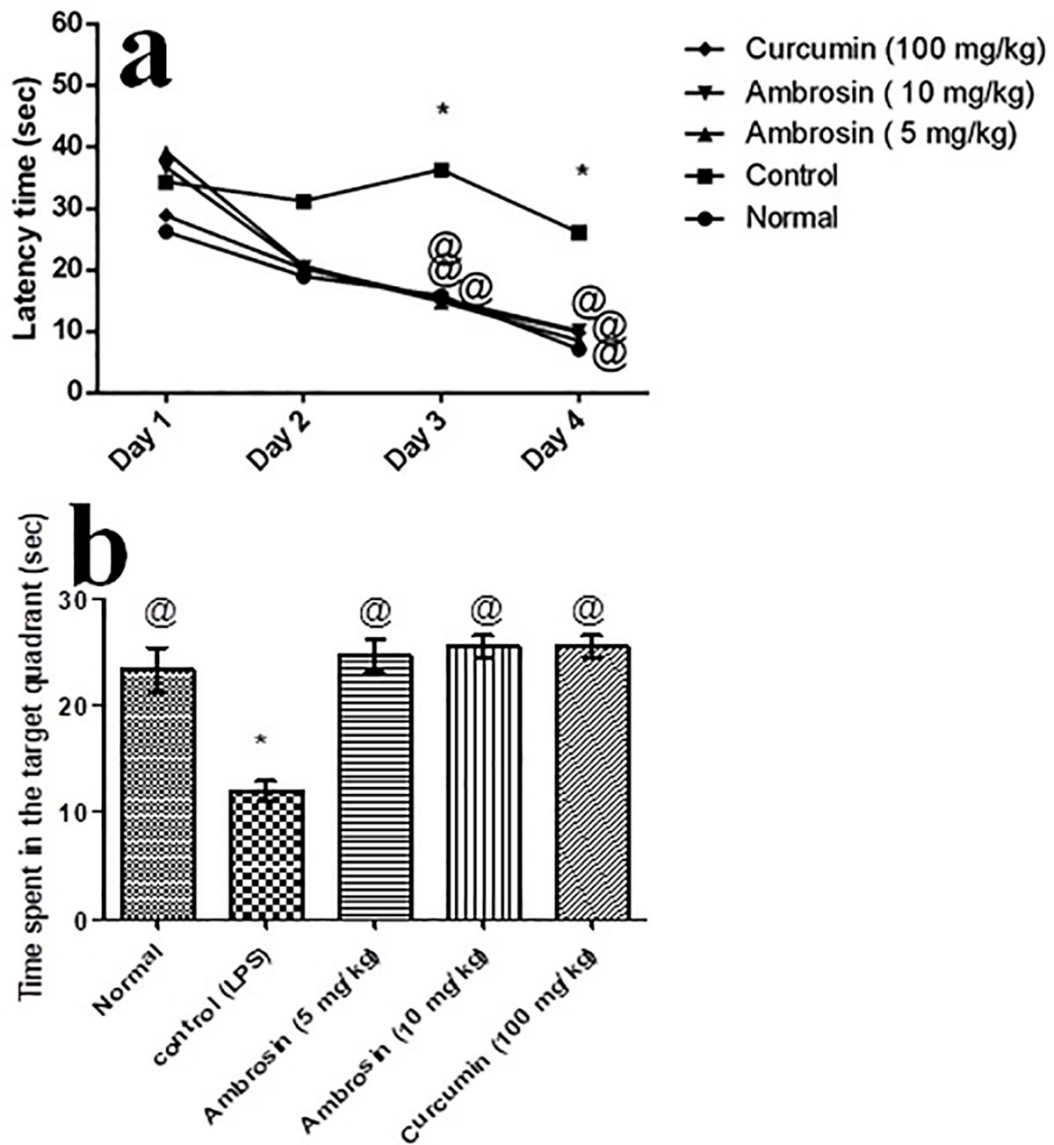
Effect of different treatments on different parameters of MWM. **a) Effect on the mean escape latency time. b) Effect on the mean time spent in the target quadrant.** Results are expressed as mean±SEM (n = 8). *Significant difference from normal group at *p*< 0.05. ^@^Significant difference from control (LPS) group at *p*< 0.05.

No significant difference in the mean escape latency time was observed between different groups on the first two days of training. However, on the 3^rd^ and 4^th^ days, LPS caused a significant prolongation in the escape latency time compared to that of the normal group **(Fig 2A, Table 2)**. This prolongation indicated a significant deterioration in the learning and spatial memory. On the contrary, curcumin and ambrosin (5 and 10 mg/kg) maintained a consistent reduction in the escape latency time similar to the pattern of the normal group (**Fig 2A) (Table 2)** indicating protection against the deterioration induced by LPS.

**Table 2:** Mean escape latency time in the MWM.

Groups	Mean escape latency time in MWM (sec)			
	Day 1	Day 2	Day 3	Day 4
**Normal**	26.27±2.26	18.98±2.59	15.87^@^±1.28	7.10^@^±0.70
**Control**	34.25±3.31	31.18±3.56	36.27*±4.26	26.11*±1.14
**Ambrosin (5 mg/kg)**	39.19±3.82	20.70±0.57	14.86^@^±1.13	8.54^@^±0.67
**Ambrosin (10 mg/kg)**	36.66±5.86	20.41±3.45	15.35^@^±1.59	10.13^@^±2.23
**Curcumin (100 mg/kg)**	28.87±2.89	20.27±3.45	14.89^@^±1.57	9.81^@^±2.22

Results are expressed as mean±SEM (n = 8).

*Significant difference from normal group at *p*< 0.05.

^@^Significant difference from control (LPS) group at *p* < 0.05.

Furthermore, LPS caused a reduction in the mean time spent in the target quadrant compared to that of normal mice, 12.20 vs 23.38 sec, indicating an impairment in the spatial memory. On the contrary, curcumin and ambrosin (5, 10 mg/kg) increased the mean time spent in the target quadrant to be 24.72, 25.59, and 25.56 sec, respectively (**Fig 2B).** The results indicated an effective halting of the detrimental effects of LPS on memory.

#### Curcumin and ambrosin ameliorated recognition memory dysfunction induced by LPS injection, performance in ORT

**Fig 3:**
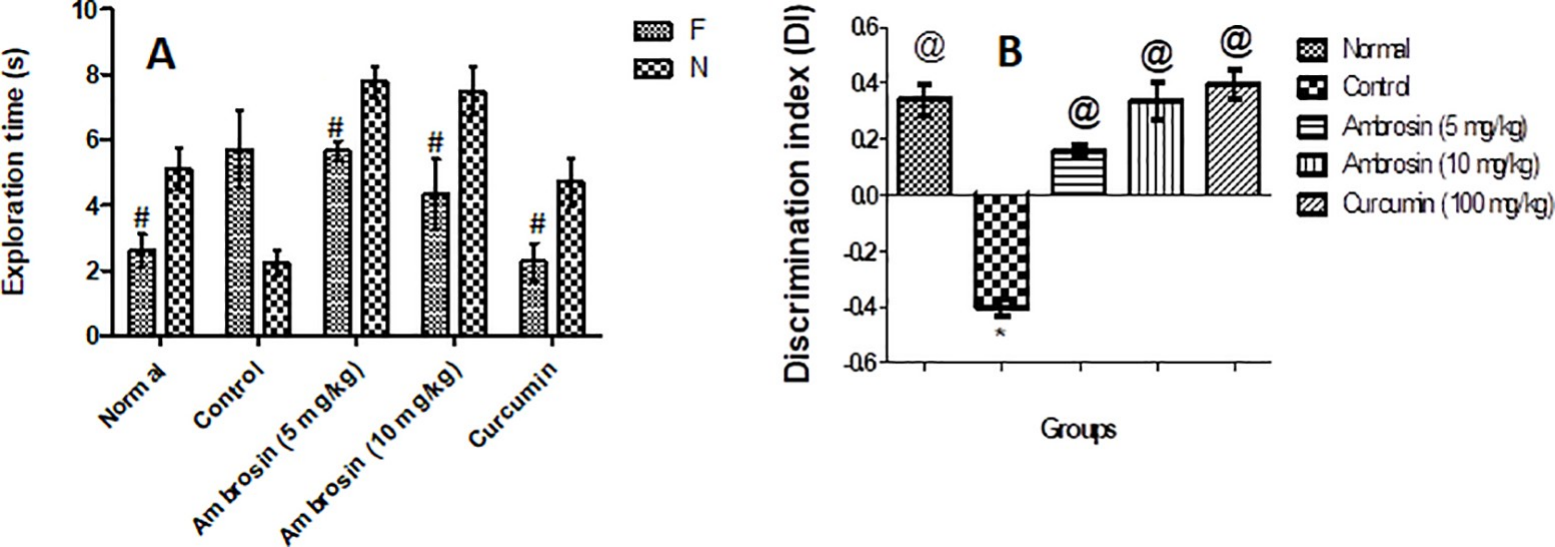
Effect of different treatments on LPS-induced memory deterioration in mice utilizing the object recognition test. **a) Exploration time of familiar (F) vs. the novel object (N). b) Discrimination index (DI)** Results are expressed as mean ±SEM (n = 8). Statistical analyses were carried out a) by using Student’s t-test, while in b) by one-way ANOVA followed by Tukey’s multiple comparison test. ^#^Significant difference versus correspondent N group at *p*< 0.05. *Significant difference from normal group at *p*< 0.05. ^@^Significant difference from control (LPS) group at *p*< 0.05.

Novel object recognition test represents a spontaneous and non-forcing memory test where mice are innately curious to explore novel objects. LPS injection deteriorated the recognition memory where mice did not show any significant difference in exploration time between novel (N) and familiar (F) objects (**Fig 3A).** However, ambrosin (5, 10 mg/kg) and curcumin (100 mg/kg) treated mice had significantly spent more time exploring N in comparison to F (**Fig 3A).** These findings were further elucidated by determination of discrimination index (DI). All mice except control had significantly discriminated N better than F (**Fig 3B).**

#### Curcumin and ambrosin reduced NF-κβp65 transcript and protein levels

**Fig 4:**
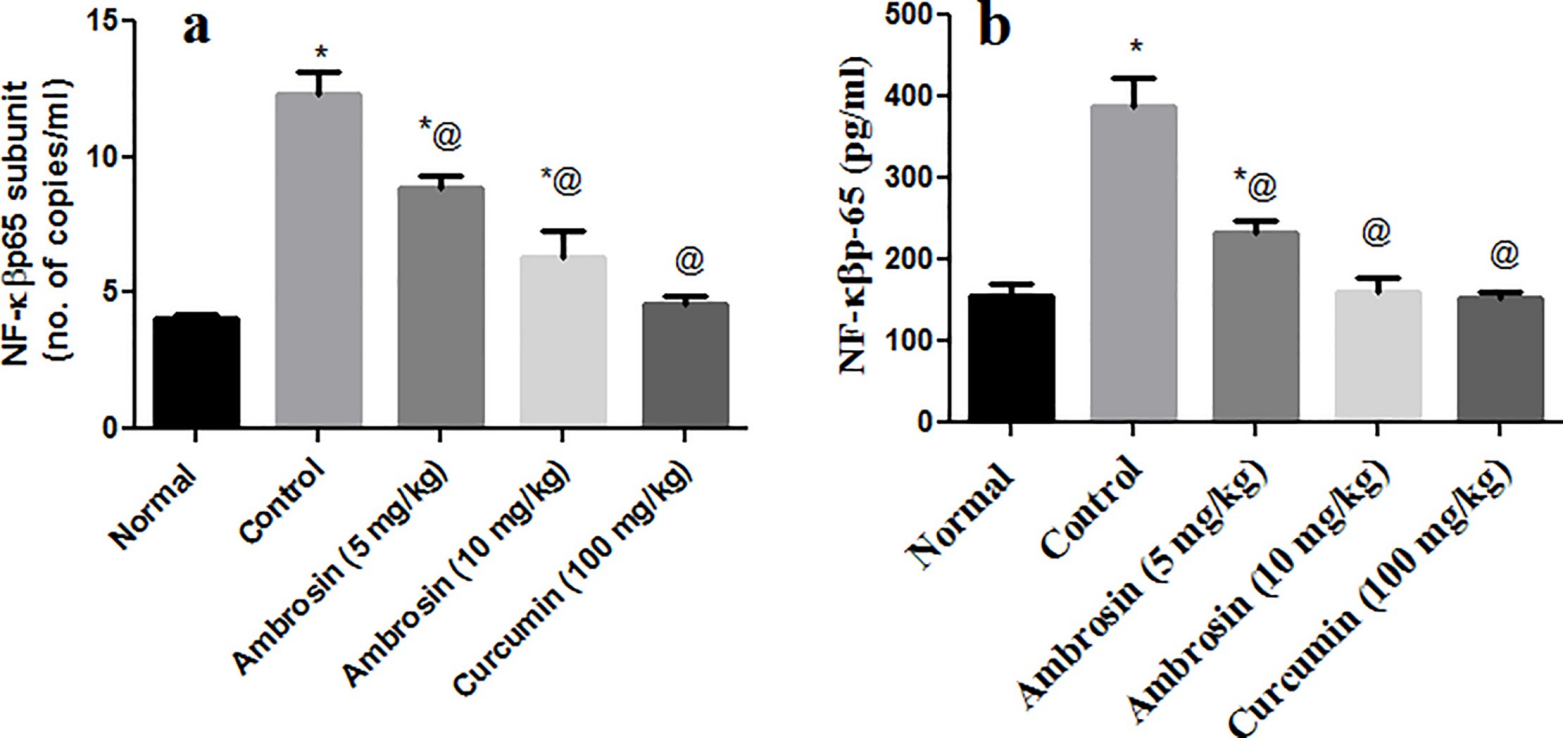
**Suppression of NF-**κβ**p65 transcript (a) and protein levels (b) by curcumin and ambrosin administration.** Data represented mean ± SEM (n = 6). Statistical analysis was performed using one-way ANOVA followed by Tukey`s multiple comparison test. *Significantly different from normal group at P<0.05. ^@^Significantly different from control group at P<0.05.

NF-κβp65 plays a malicious role in neuroinflammation, Aβ deposition and neuronal apoptosis [26, 27]. Therefore, it is a novel target in AD therapy. Systemic administration of LPS increased the expression of NF-κβp65 significantly (3 folds) and significantly increased its protein level (2.5 folds) (**Fig 4A and 4B).** Curcumin had halted the upsurge of NF-κβp65, maintaining it almost normal on both transcript and protein levels. Ambrosin had significantly halted the expression of NF-κβP65 in a dose-dependent manner whereas larger dose (10 mg/kg) had better results and kept NF-κβp65 at levels significantly lower than the control group. Inhibition of expression was reflected in the significant reduction of NF-κβp65 protein levels which was not significantly different from normal group.

#### Curcumin and ambrosin attenuated neuroinflammation by reducing pro-inflammatory cytokines and enzymes

**Fig 5:**
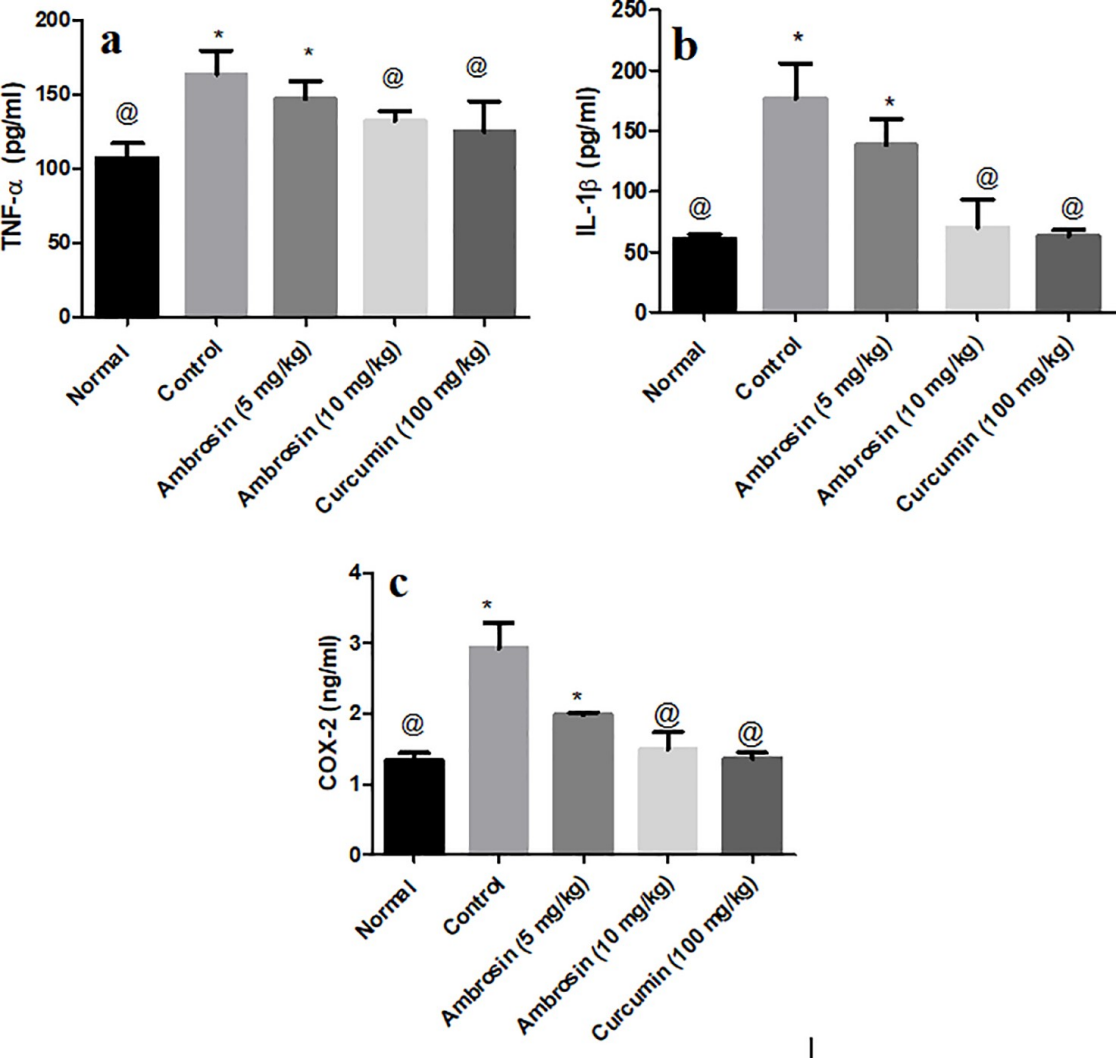
**Curcumin and ambrosin alleviated neuroinflammation by inhibition of repression of pro-inflammatory cytokines, *viz***.**, TNF-**α **(a), IL-1β and enzymes, *viz*., COX-2 (c).** Data represented mean ± SEM (n = 6). Statistical analysis was performed using one-way ANOVA followed by Tukey`s multiple comparison test. *Significantly different from normal group at P<0.05. ^@^Significantly different from control group at P<0.05.

Peripheral inflammation activates microglia in the brain and stimulate it to secrete pro-inflammatory cytokines, such as IL-1β and TNF-α [28]. Furthermore, NF-κβp65 induces upregulation of COX-2 enzyme [3]. LPS treatment resulted in significant increase in the protein levels of TNF-α (1.5 folds), IL-1β (3.5 folds) and COX-2 (3 folds) (**Fig 5)**. Both curcumin and ambrosin (10 mg/kg) had significantly reduced the elevated levels back to normal values (*p* < 0.05). Nevertheless, ambrosin at low dose (5 mg/kg) resulted in significant reduction of the estimated pro-inflammatory mediators. These results highlighted the potent anti-inflammatory activities of both curcumin and ambrosin (10 mg/kg).

#### Curcumin and ambrosin reduced the production of Aβ by reducing BACE1 enzyme levels

**Fig 6:**
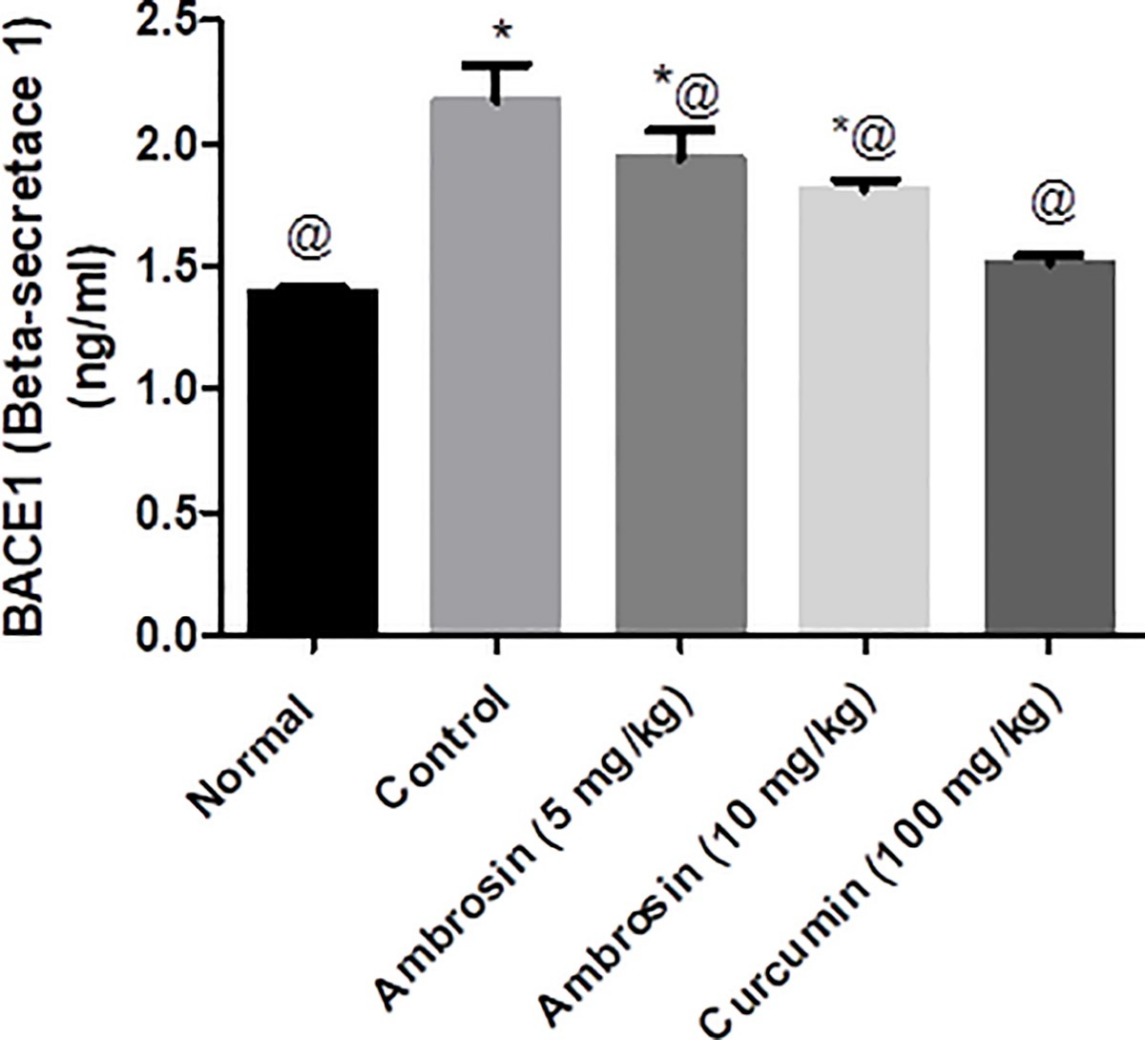
Curcumin and ambrosin abated the elevation in BACE1 levels after LPS administration. Data represented mean ± SEM (n = 6). Statistical analysis was performed using one-way ANOVA followed by Tukey`s multiple comparison test. *Significantly different from normal group at P<0.05. ^@^Significantly different from control group at P<0.05.

BACE1 plays a central part in production and deposition of Aβ where it performs the first cleavage step of APP (Amyloid Precursor Protein). Excessive production and deposition of Aβ plaques was correlated to elevated levels and activity of BACE1. BACE1 levels were increased significantly after LPS treatment (50% increment) (**Fig 6).** Curcumin effectively decreased BACE1 protein levels to levels similar to normal levels. Ambrosin caused a dose-dependent reduction of BACE1 levels which were significantly lower than levels observed in the control untreated group.

#### Curcumin and ambrosin abated the elevation in iNOS levels production

**Fig 7:**
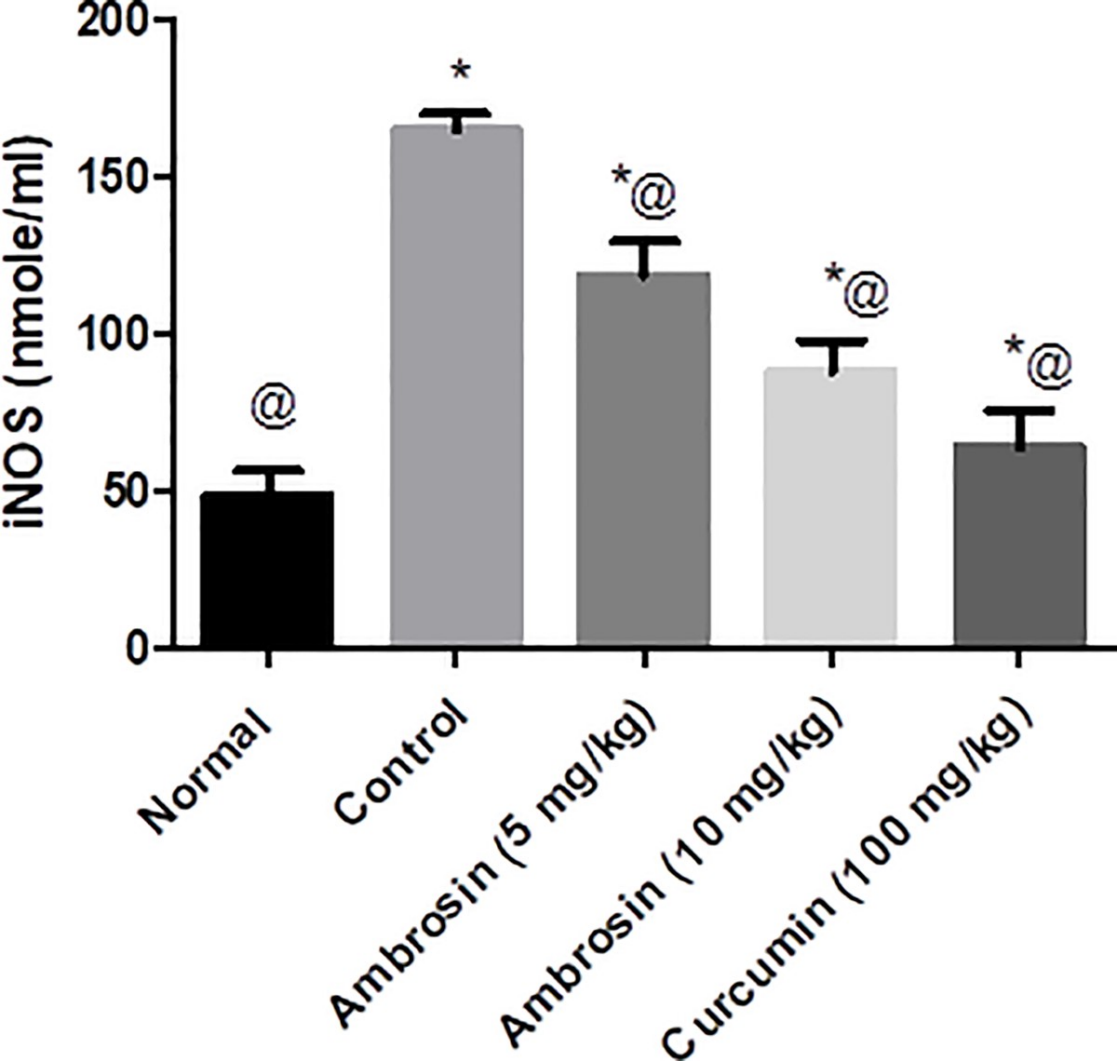
Curcumin and ambrosin abated the elevation in iNOS levels after LPS administration. Data represented as mean ± SEM (n = 6). Statistical analysis was performed using one-way ANOVA followed by Tukey`s multiple comparison test. *Significantly different from normal group at P<0.05. ^@^Significantly different from control group at P<0.05.

iNOS plays a significant role in production of noxious NO which further aggravates Alzheimer manifestation [29]. Its protein levels were increased after LPS treatment (3 folds) (**Fig 7)**. Curcumin and ambrosin (5 and 10 mg/kg) had significantly reduced the elevation in iNOS levels when compared to control untreated group. Curcumin was the most active followed by ambrosin (10mg/kg) then ambrosin (5 mg/kg).

#### Curcumin and ambrosin attenuated Aβ deposition and neuronal death

**Fig 8:**
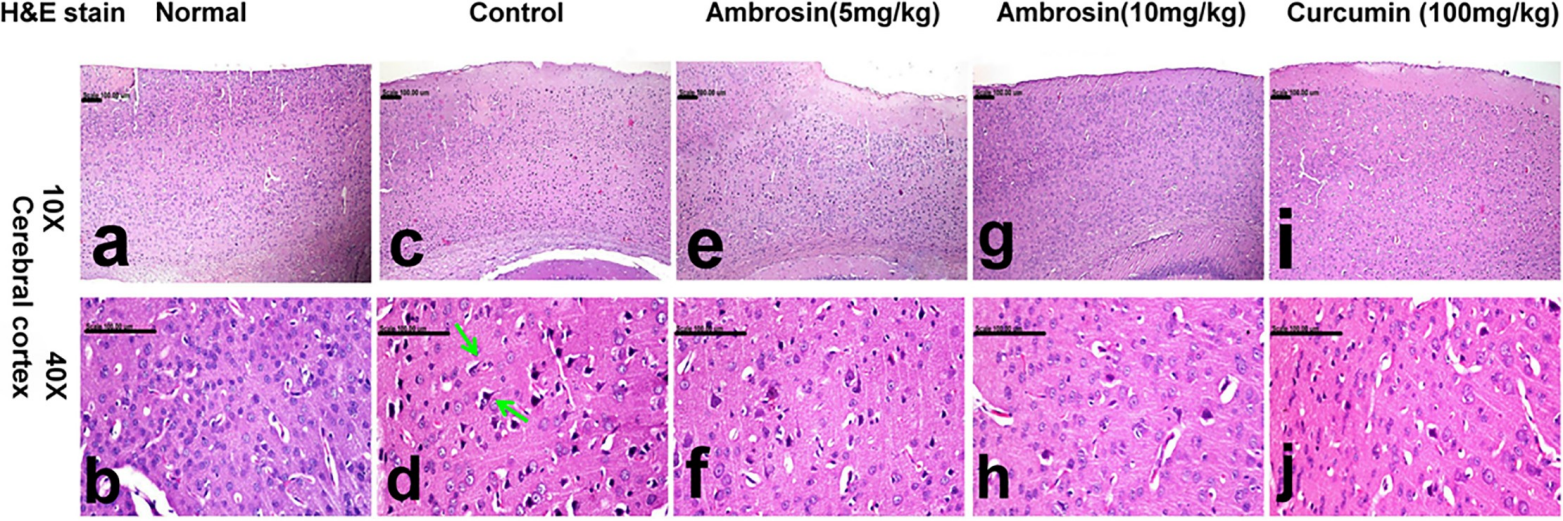
Histopathological lesions in the cerebral cortex of mice (magnifications: 10X, 40X). The photomicrograph illustrates the following groups: (a, b) normal mice showing normal cerebral cortex, (c, d) control mice showing neuronal degeneration associated with presence of neurofibrillary tangles (arrow) and amyloid plaque with eosinophilic core, (e, f) Ambrosin (5 mg/kg/day) treated mice showing neurons bearing tangles and gliosis, (g, h) Ambrosin (10 mg/kg/day) treated mice demonstrating lower number of degenerated neurons, and (i, j) Curcumin treated mice showing scattered degenerated neurons. (Haematoxylin and eosin stain, (H&E stain)).

**Fig 9:**
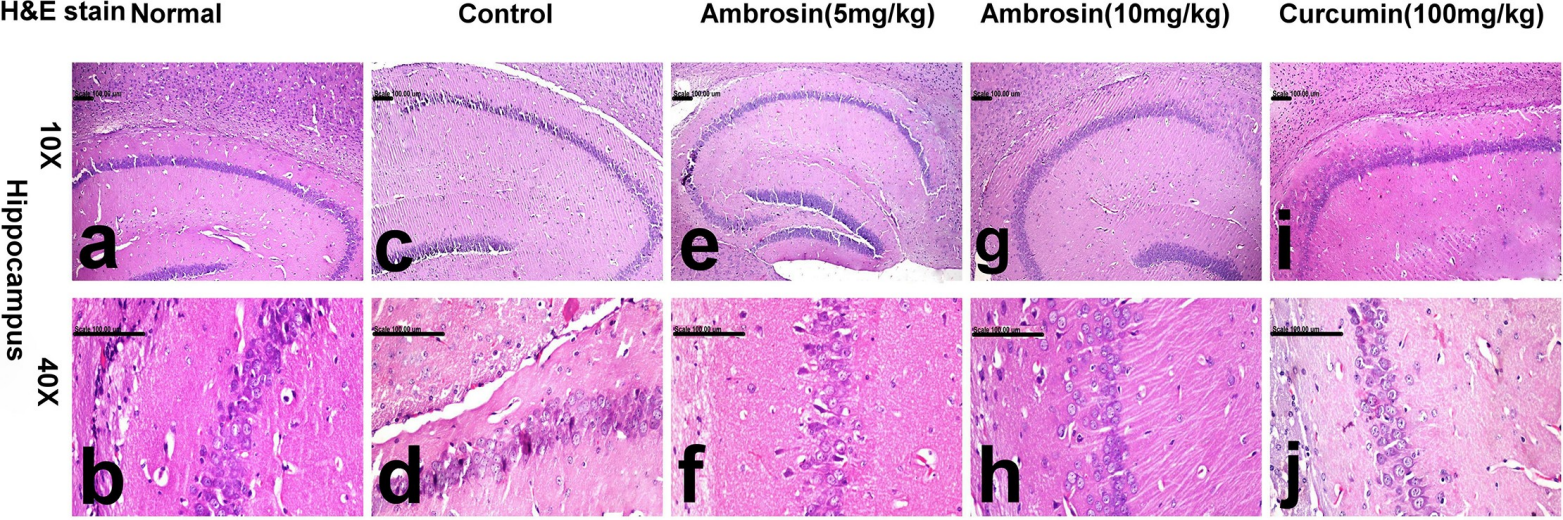
Histopathological lesions in the hippocompus of mice (magnifications: 10X, 40X). The photomicrograph illustrates the following groups: (a, b) normal mice showing normal hippocampal neurons, (c, d) control mice showing extensive degeneration of pyramidal neuronal cells, (e, f) Ambrosin (5 mg/kg/day) treated mice showing decreased number of degenerated neurons, (g, h) Ambrosin (10mg/kg/day) treated mice showing degeneration of individual pyramidal neurons, and (i, j) Curcumin treated mice showing normal neuronal cells. (Haematoxylin and eosin stain, (H&E stain)).

**Fig 10:**
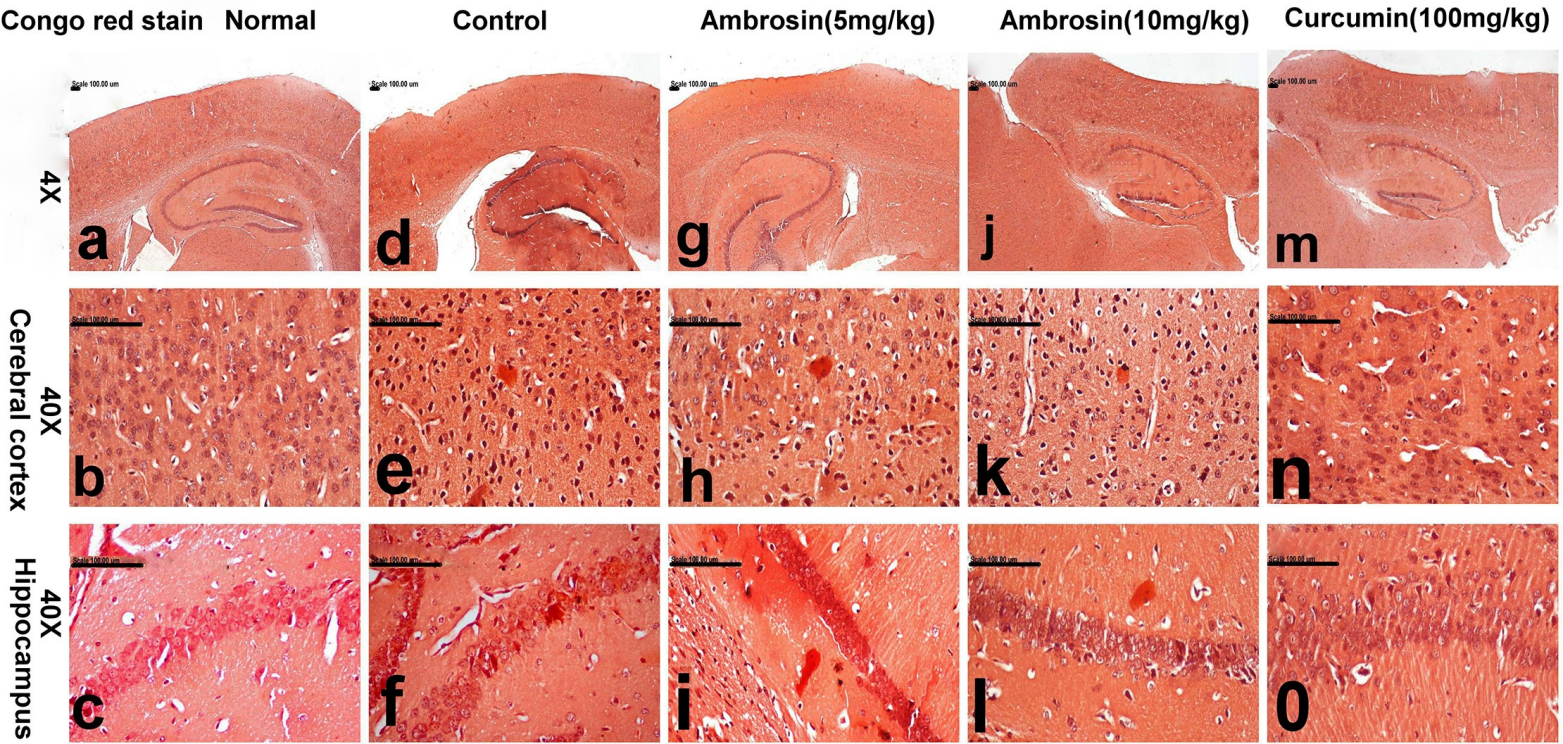
Brain sections of mice stained with Congo red stain for the demonstration of amyloid plaques (magnifications: 4X, 10X, 40X). The photomicrograph illustrates the following groups: (a-c) normal mice showing normal cerebral cortex (b) and hippocampus (c), (d-f) control mice showing abundant red-stained amyloid plaques in the cerebral cortex (e) and hippocampus (f), (g-i) Ambrosin (5mg/kg/day) treated mice showing decreased number of amyloid plaques in the cerebral cortical (h) and hippocampal tissues (i), (j-l) Ambrosin (10mg/kg/day) treated mice showing few amyloid plaques in cerebral cortex (k) and hippocampus (i),(m-o) Curcumin treated mice showing small amyloid plaques in cerebral cortex (n) and no amyloid plaques in hippocampus (o). (Congo red stain).

Histopathological examination of brain tissues gave further confirmation of the protective actions of curcumin and ambrosin against AD. The most striking observations were the number of surviving neurons and quantity of Aβ depositions (**Table 3).** Brain of normal mice showed normal histology with normal cerebral cortex (**Fig 8A and 8B)** and hippocampus (**Fig 9A and 9B)** with no amyloid deposition (**Fig 10A–10C).** In contrast, brain of control mice revealed extensive neuronal degeneration that was characteristically demonstrated in the inner granular, ganglionic and multiform layers of the cerebral cortex associated with activation of glia cells in addition to presence of neurofibrillary tangles. These tangles appeared in pyramidal neurons as slightly basophilic threads on one side of the nucleus (**Fig 8C and 8D).** Abundant amyloid plaques with eosinophilic core were primarily demonstrated in the entorhinal cortex (Fig 10E) and hippocampus. Hippocampus of this group revealed extensive degeneration of pyramidal neuronal cells with marked decreased number of surviving neuron (23.33±1.45) compared to normal hippocampus (56.66±2.90) in addition to deposition of amyloid plaques (**Fig 9C and 9D)** that appeared red in Congo red stained sections (**Fig 10F).**

**Table 3:** Quantification of surviving neurons in the hippocampal CA1 region and quantification of amyloid plaques found by histochemical examination of brains of tested groups.

Group*	Number of surviving neurons in the hippocampal CA1 region (high power field)*	Number of amyloid plaques (high power field)
**Normal**	56.66^d^±2.90	————-
**Control**	23.33^a^±1.45	6.20^a^±0.29
**Ambrosin (5mg/kg)**	35.66^b^±4.63	2.50^b^±0.30
**Ambrosin (10 mg/kg)**	44.33^c^±1.20	0.90^c,d^±0.23
**Curcumin**	53.33^c,d^±4.17	0.50^d^±0.16

* Data represented as means ± SEM. No significant difference presents between groups having same letter.

Treatment with ambrosin showed marked attenuation of the histopathological lesions in a dose-dependent manner. Slight decrease in the number of neurons bearing tangles and gliosis was demonstrated in the cerebral cortex of ambrosin (5 mg/kg/day) (**Fig 8E and 8F).** Additionally, slight increment of surviving neurons (35.66±4.63) and reduction of degenerated ones were traced in the hippocampus of this group (**Fig 9E and 9F)** in addition to reduced number of amyloid plaques in the cerebral cortical and hippocampal tissues (**Fig 10G–10I).** Ambrosin at dose level (10 mg/kg) had a more significant better results and close similar to curcumin ones. There was significant increment of the surviving neurons of cerebral cortex (**Fig 8G and 8H)** and hippocampus (44.33±1.20) (**Fig 9G and 9H)** in addition to decreased number of amyloid plaques in the cerebral cortical and hippocampal tissues (**Fig 10J–10L,** respectively). The most prominent improvement was recorded in curcumin group, in which most neurons of cerebral cortex (**Fig 8I and 8J)** and hippocampus appeared normal with scattered degenerated neurons (**Fig 9I and 9J).** Moreover, the amyloid plaques were fewer and small in size compared to other treated groups (**Fig 10M–10O).**

### Immunohistochemistry

The quantitative assay of the optical density (OD) of the immune reactivity of COX-2, CD68 and cleaved caspase-3 recorded in the brain of demented and treated mice was illustrated in **Table 4**.

**Table 4:** Quantification of pro-inflammatory and pro-apoptotic markers traced by immunohistochemical examination of brains of tested groups.

Group*	COX2 (OD)	CD68 (OD)	Cleaved caspase-3 (OD)
**Normal**	0.19^a^±0.00	0.12^a^±0.00	0.13^a^±0.01
**Control**	0.48^d^±0.01	0.30^d^±0.01	0.46^d^±0.01
**Ambrosin (5 mg/kg)**	0.38^c^±0.01	0.20^c^±0.01	0.37^c^±0.01
**Ambrosin (10 mg/kg)**	0.31^b^±0.02	0.15^b^±0.01	0.20^b^±0.01
**Curcumin**	0.30^b^±0.01	0.13^a,b^±0.01	0.19^b^±0.05

* Data represented as means ± SEM. No significant difference presents between groups having same letter.

#### Curcumin and ambrosin reduced COX-2 immunoreactivity

**Fig 11:**
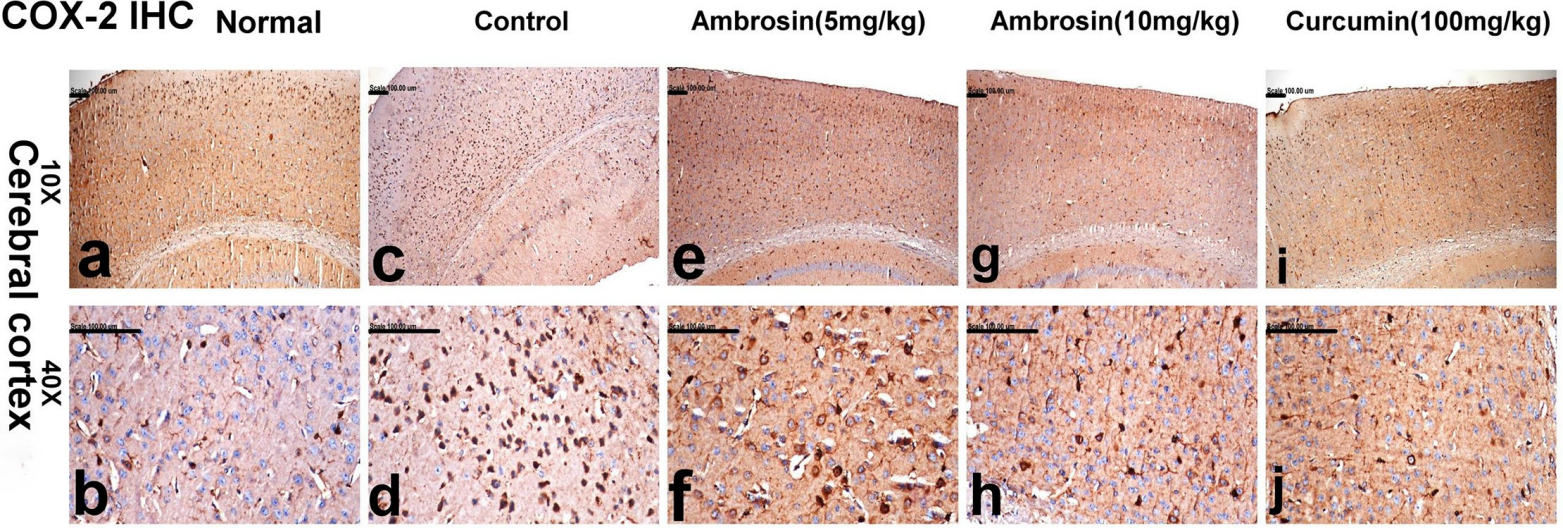
COX-2 immune-stained neurons in the brain sections of mice (magnifications: 10X, 40X). The photomicrograph illustrates the following groups: (a, b) normal mice showing insignificant COX-2-immune positive cells, (c, d) control mice showing significant increase of COX-2 expression in the cerebral cortical neurons, (e, f) Ambrosin (5 mg/kg/day) treated mice showing reduction of COX-2 expression, (g, h) Ambrosin (10 mg/kg/day) treated mice showing COX-2 expression in fewer numbers of neurons, and (i, j) Curcumin treated mice showing individual COX-2 immune-stained cells. (COX-2 immunohistochemical stain).

Coinciding with ELISA results, COX-2 transcripts were elevated in the mice of control group in comparison to normal and treated groups. Significant increase of COX-2 immune reactivity was recorded in the control mice (0.48±0.01) compared to the normal group (0.19±0.00). The immune reactivity was mainly demonstrated in the ganglionic and multiform neurons of cerebral cortex (**Fig 11C and 11D).** Marked reduction of COX-2 immune reactivity was recorded in ambrosin (5mg/kg) (0.38±0.01) (**Fig 11E and 11F),** compared to the control group. On the other hand, significant reduction of COX-2 immune reactivity was demonstrated in ambrosin (10 mg/kg) (0.31±0.02) (**Fig 11G and 11H)** and curcumin (0.30±0.01) (**Fig 11I and 11J)** with no significant difference between them.

#### Curcumin and ambrosin reduced CD68 immunoreactivity

**Fig 12:**
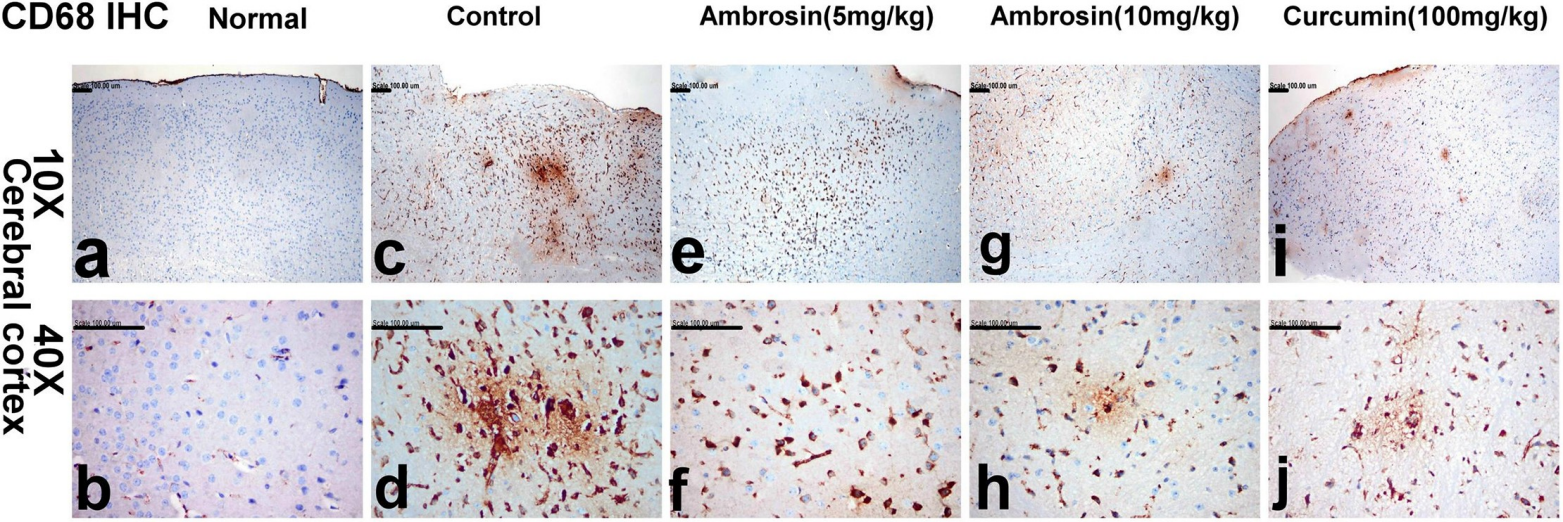
CD68 -immune reactive microglia in the brain sections of mice (magnifications: 10X, 40X). The photomicrograph illustrates the following groups: (a, b) normal mice showing sparse weakly CD68 positive immune stained cells, (c, d) control mice showing significant increase of CD68 immune stained microglia surrounding amyloid plaques, (e, f) Ambrosin (5mg/kg/day) treated mice showing reduction of CD68 immune stained microglia, (g, h) Ambrosin (10mg/kg/day) treated mice showing small focal aggregates of CD68 immune stained microglia, and (i, j) Curcumin treated mice showing few CD68 immune stained microglia. (CD68 immunohistochemical stain).

CD68 is a marker for microglia activation in many studies [28]. Sparse weakly CD68 positive immune reactive cells were demonstrated in the normal group (0.12±0.00) (**Fig 12A and 12B).** However, significant increase of CD68 immune reactive microglia was observed in the control group (0.30±0.01) (**Table 4).** The cells appeared hypertrophied with enlarged cell bodies surrounding amyloid plaques (**Fig 12C and 12D).** Reduced CD68 immune reactivity was recorded in ambrosin (5 mg/kg) (0.20±0.01) (**Fig 12E and 12F)** being significantly different from the control group. Prominent reduction of CD68 immune reactivity was recorded in ambrsosin (10 mg/kg) (**Fig 12G and 12H)** and curcumin groups (0.15±0.01 and 0.13±0.01, respectively) in which small focal aggregates of CD68 immune reactive microglia were demonstrated (**Fig 12I and 12J), respectively.** The reduction was in accordance with reduced Aβ deposition and reduced neuronal death.

#### Curcumin and ambrosin decreased caspase-3 immunoreactivity

**Fig 13:**
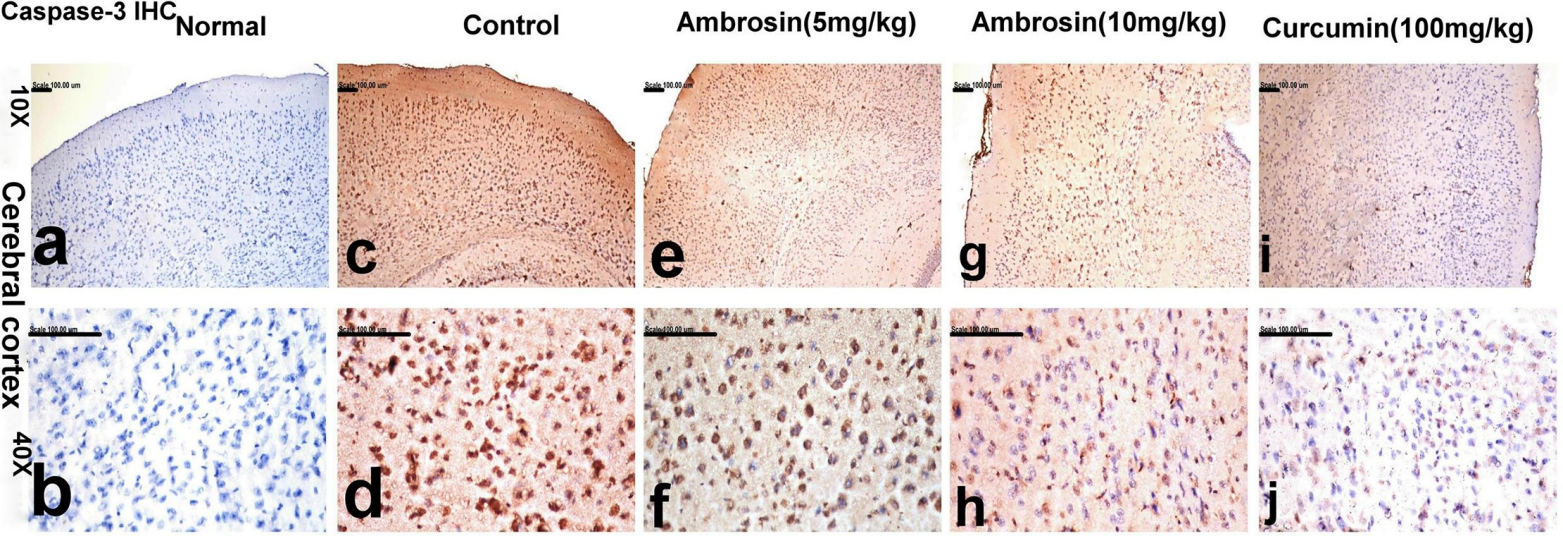
Cleaved caspase-3 immune reactive neurons in the brain sections of mice (magnifications: 10X, 40X). The photomicrograph illustrates the following groups: (a, b) normal mice showing no caspase-3 immune reactive cells, (c, d) control mice showing significant increase of caspase-3 immune reactivity, (e, f) Ambrosin (5 mg/kg/day) treated mice showing reduction of caspase-3 immune reactivity, (g, h) Ambrosin (10 mg/kg/day) treated mice showing significant reduction of caspase-3 immune reactivity, and (i, j) Curcumin treated mice showing weak caspase-3 immune reactivity. (Cleaved caspase-3 immunohistochemical stain).

Activated (cleaved) caspase-3 immune reactivity was correlated with synaptic loss and neurodegneration in AD [30]. Negligible caspase-3 immune reactive cells were observed in the normal group (0.13±0.01) (**Fig 13A and 13B).** However, significant increase of these reactive cells was demonstrated in the cerebral neurons of control mice (0.46±0.01) (**Fig 13C and 13D).** Treatment with ambrosin revealed reduction of caspase-3 immune reactivity in a dose-dependent manner (0.37±0.01 and 0.20±0.01, respectively) (**Fig 13E–13H), respectively.** Weak immune reactivity of caspase-3 was recorded in curcumin groups (0.19±0.05) (**Fig 13I and 13J).**

### Molecular docking to NF-κβp65

**Fig 14:**
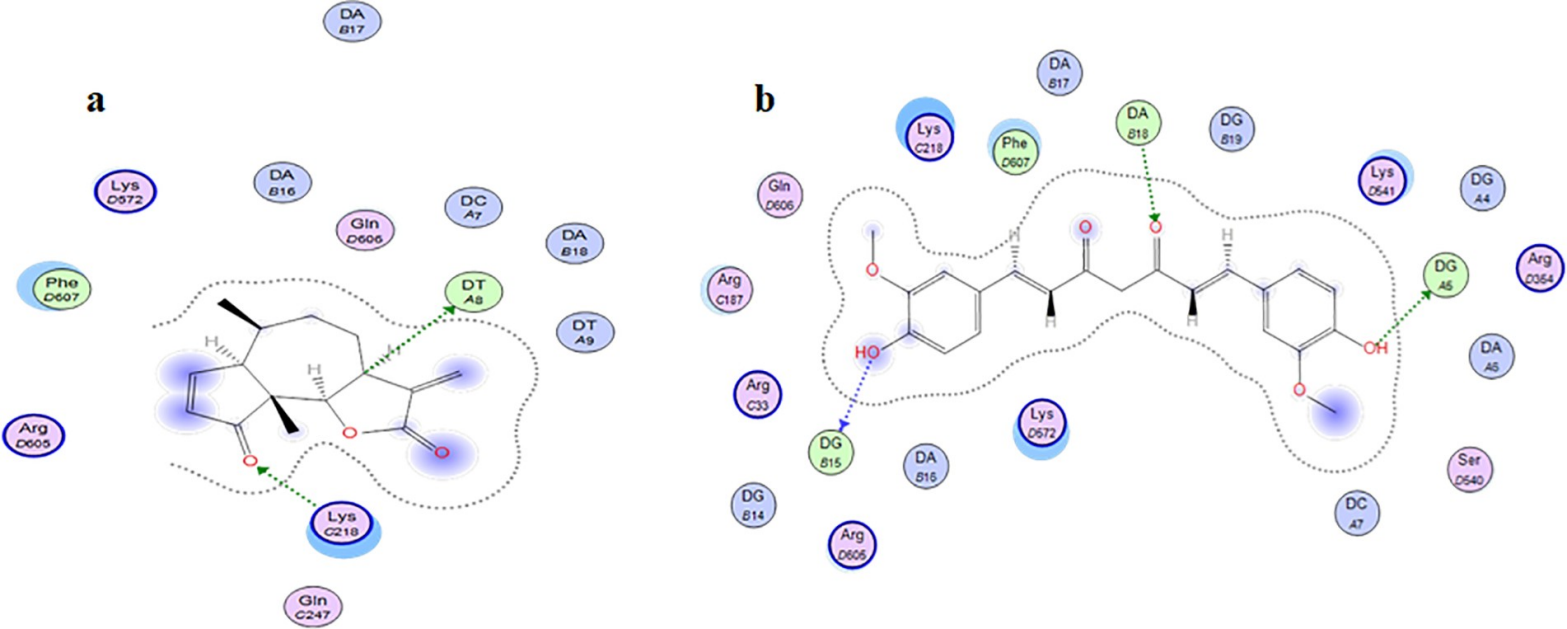
**Binding modes of ambrosin (a) and curcumin (b) showing polar interactions with DNA bases within NF-ҡβp65.** DNA nucleotides are indicated by light green colors. Amino acids residues are blue pink in color. Hydrogen bonds are indicated as dotted arrows Hydrophobic interactions are highlighted in violet circles.

Although ambrosin was found to inhibit NF-κβp65 *in vitro* [12], no docking simulation study was performed to explore the its possible interactions, similary, curcumin was never docked to NF-κβp65. The present study reports the docking scores and interaction modes (**Table 5**). Unlike the known reports indicating that sesquiterpene lactones binds covalently to Cysteine residues in their targets [31]. It was shown that ambrosin can form two H-bonds with amino acid residues in the active site. H-bond between carbonyl group at position 4 and lysine (218) might explain the higher reactivity of ambrosin when compared to other pseudoguainolides having one carbonyl function [12] (**Table 5, Fig 14A)**. Previous studies highlighted the essentiality of lactone ring for the activity. Hereto, the current model highlighted the hydrophobic interactions around the lactone ring and the presence of H-bond between H-7 and thymidine nucleotide. Direct interaction between curcumin and NF-κβp65 was not previously studied. Curcumin can form more H bonding than do ambrosin because of more oxygenation pattern (**Table 5, Fig 14B)**. It can bind more stably to both the DNA and the protein. This explains why curcumin has lower interaction energy score (London–dG). Both types of binding interaction through H-bonding and/or hydrophobic binding demonstrates clearly reversible inhibition of NF-ҡβ‎p65 activation.‎ Further experimental confirmation of the current docking study is beyond the scope of the current study.

**Table 5:** Docking Scores and interaction modes of Curcumin and Ambrosin with NF-ҡβp65 (PDB ID: 1VKX).

Compound	-dG (Pose #)	Interaction	Distance (Å)	E(kcal/ mol)
Curcumin	**-8.2526 (2)**	DG-5(C) H-donor	3.37	-1.1
		DG-15(D) H-donor	3.07	-2.3
		DA-18(D) H-acceptor	3.09	-0.9
	**-7.4516 (23)**	DG-5(C) H-donor	3.12	-2.3
		ARG-187(A) H-donor	3.32	-1.4
		DA-17(D) H-acceptor	2.89	-2.1
Ambrosin	**-4.0236 (11)**	DT-8(C) H-donor	3.16	-0.8
		LYS-218(A) H-acceptor	2.57	-1.1

## Discussion

As speculated, nonpolar NF-κβ inhibitors can ameliorate systemic inflammation induced dementia in a comparable degree to curcumin. Equivalent efficacy was attainable using ambrosin at much lower dose (one tenth). Hereto, *in silico* studies speculated that ambrosin had better BBB permeability compared to curcumin due to its lower log P and TPSA values (**Table 2).** However, poor bioavailability of curcumin was attributed to factors other than lipophilicity, namely, instability in alkaline intestinal content, intracellular accumulation, degradation during transport, rapid metabolism and excretion [32]. Therefore, the promising pharmacokinetic properties of ambrosin should be assessed by further *in vivo* studies. In the current study, curcumin and ambrosin were administered intraperitoneally to determine their efficacy regardless their oral bioavailability.

LPS-induced memory deficit represents an important model for the pre-clinical evaluation of nutraceuticals`efficacy in alleviation of AD [33]. The systemic inflammation induced by administration of LPS causes a cascade of central neuroinflammation and neurodegeneration in brain. These events are mediated by activation of microglia and subsequent release of pro-inflammatory cytokines. This aggravates the progression of neuroinflammation and in turn activates the formation and deposition of Aβ these events lead to a vicious cycle of neurodegenration and deposition of Aβ plaques. Eventually, these cycles result in deterioration of behavioral, cognitive, learning and memory functions [33]. NF-κβ plays a central role in this vicious cycle by regulating the transcription of many pro-inflammatory cytokines and mediating the Aβ production as well as Aβ-induced toxicity [3]. Therefore, the present work focused on determining the inhibition NF-κβ and its downstream targets.

In the present study, systemic LPS administration resulted in upregulation of NF-κβp65 transcript and protein levels **(Fig 4)**. The balance between c-rel containing dimers and the p65:p50 dimer of NF-κβ preserves neuronal plasticity, synaptic activity and neuroprotection. However, under pathological conditions the excessive activation of P65:p50 dimer disrupts this balance to enhance transcription of cytokines, pro-inflammatory and apoptotic factors leading to neuroinflammation and neurodegeneration [4, 26]. The upsurge of NF-κβp65 was attenuated by curcumin and ambrosin. Interestingly, these inhibitors had quite different mechanisms of action.

Curcumin inhibits NF-κβ by inhibiting the degradation of Iκβα [34], hence this inhibited the activation of NF-κβ and its translocation to the nucleus. In contrast, ambrosin has two mechanisms of action. First, ambrosin directly inhibits the interaction between NF-κβp65 and DNA. The α-methylene-γ-lactone moiety **(Fig 1)** reacts as Michael acceptor and directly reacts with thiol group of cyteine-38 and cysteine-120 in P65 subunit rendering the molecule unable to bind to DNA [12, 31, 35, 36]. In this aspect, ambrosin is more active than other STL because of presence of another reactive center which is the α,β unsaturated carbonyl group **(Fig 1)** [12, 36]. The second mechanism was similar to that of curcumin, through inhibition of Iκβ degradation [37]. However, the inhibition of Iκβ degradation occurred at high concentrations of STL and this pathway was presumed to contribute minorly to inhibition of NF-κβ by STL [31, 38]. The aforementioned mechanisms targeted either the activation step of NF-κβ or rendering NF-κβ inactive to bind DNA. Co crystallization of NF-κβp65 with an inhibitor was not performed before. The presented molecular docking study gave insights that curcumin and ambrosin can interact with DNA-NF-κβp65 complex and establish a stable binding. However, further experimental studies should be performed to confirm such a hypothesis.

Apart from direct interaction or inhibition of activation of NF-κβ, the current study showed that both curcumin and ambrosin had downregulated the expression of NF-κβp65 (**Fig 4)**. That inhibition was reflected in alleviation of three aspects of AD, namely, neuroinflammation, amyloidogenesis and oxidative stress.

Regarding neuroinflammation, ambrosin and curcumin had effectively inhibited the downstream pro-inflammatory mediators, namely, COX-2, TNF-α and IL-1β **(Fig 5)**. Sesquiterpene lactones from chicory roots inhibited expression of COX-2 by inhibiting NF-κβ [39]. TNF-α plays an important role in mediating the neuroinflammation due to systemic LPS administration [16]. STL from *Saussurea* attenuated the release of TNF-α produced by RAW macrophages upon stimulation by LPS [40]. It was demonstrated that α-methylene-γ-lactone moiety was essential for such an activity. Ambrosin and curcumin had reduced IL-1β levels. Interfering with IL-1β signaling was found to rescue cognition, attenuates Tau pathology and restore neuronal β-catenin pathway [41]. Several STL inhibited the release of cytokines and lymphocyte proliferation through inhibition of NF-κβ activation [42].

Regarding amyloidogenesis, ambrosin and curcumin had effectively reduced the deposition of Aβ plaques as quantified by histopathological examination (**Table 3) (Fig 10).** This could be attributed to decrease in BACE1 levels (**Fig 6)**, an outcome to inhibition of NF-κβp65. BACE1 promoter region contains two responsive elements for NF-κβ [43]. Even the upregulation of BACE1 due to elevated Aβ or elevated wild-type or Swedish mutated APP is mediated by NF-κβ signaling [3]. This is the first report that STL and ambrosin could play a role in inhibition of amyloidgenesis.

The hallmark of alleviation of neuroinflammation, Aβ deposition and microglia activation was further highlighted in reduction of expression of CD68 (**Table 4) (Fig 12).** Expression of CD68 correlates with the phagocytosis activity of glial cells and their role in clearance of amyloid deposits as well as cellular debris. The expression was found to correlate positively to Aβ deposition and dementia. Its levels were upregulated during clearance of Aβ and clearance of cellular damaged material [44]. Hence, reduction in activation of CD68 microglia was a reflection for reduced amyloidgenesis and reduced neurotoxicity mediated by ambrosin and curcumin.

Regarding oxidative stress, it is quite evident that NF-κβ is a redox sensitive transcription factor [27] where NF-κβ and iNOS pathways along with mitochondrial dysfunction are the three most disturbed pathways [45]. Elevated NO levels induce formation of nitrated Aβ which have detrimental structural and functional effects on brain [29]. Ambrosin and curcumin had effectively abated iNOS (**Fig 7).** Finally, the neuroprotective activity of curcumin and ambrosin were reflected in reduced cleaved caspase-3 (**Table 4 and Fig 13).**

Natural products are known for their multi-targeting nature. Inhibition of NF-κβ is not the sole mechanism of action of curcumin. Curcumin was found to interact with several other targets [6]. All these effects add to its potential and activity. In contrast, although STL have not been so exhaustively studied as curcumin, they have great potential. Damsin and neoambrosin, the isomers of ambrosin, were shown to inhibit STAT and p-glycoproteins [46]. Some STL inhibit MAPK and protein tyrosine kinase [38].

STL are diverse in structure but retaining the essential pharmacophore moiety responsible for activity [38]. We hope that investigation of STL can contribute to its repurposing in AD treatment. Recently, few studies could be traced in this aspect. STL from *Hedyosmum brasiliense* attenuated memory impairment induced by i.c.v. administration of Aβ_1–42_ [47]. Anticholinesterase activities of some STL were evaluated [48].

‎In this preliminary phase, ameliorative actions of ambrosin were studied on the whole brain homogenate level. However, the next phase will explore which brain cell types are targeted by ambrosin. Our future plans will explore four aspects. First, the *in vivo* pharmacokinetics properties of ambrosin are to be assessed. Second, ameliorative actions induced by ambrosin on specific cell types are to be investigated. Third, the efficacy of ambrosin is to be evaluated in different AD animal models to overcome the heterogeneity of the disease. Fourth, other STL are to be tested. A good candidate for that would be parthenolide, the main component of feverfew which has been utilized for centuries as antimigraine, quite an indication about its oral bioavailability. In the current study, ambrsoin was as effective as curcumin but at much lower dosage. In clinical trials, curcumin was effective in dose range 0.6–3.6 g daily [49]. However, not all patients can afford such high doses daily, especially in geriatrics. Therefore, the aim of the current study was to examine the potential of ambrosin as an additional, not a better, candidate for the currently known AD therapeutics.

## Conclusion

The current study evaluated the potential of ambrosin as a remedy for AD *via* inhibition of NF-κβp65 and its downstream signals. Ambrosin is predicted to have a good oral bioavailability and BBB permeability as predicted by *in silico* studies. It had effectively inhibited neuroinflammation, amyloidgenesis and neurone death as demonstrated from the behavioral, molecular and immunhistochemical experiments. Its activity was comparable to curcumin, the forerunner of natural products as AD remedy. Further studies are required to explore different pharmacokinetic and pharmacological properties of ambrosin and to introduce it as an additional candidate remedy for AD as many investigated natural polyphenols.

## Supporting information

S1 Fig HPLC chromatogram of isolated ambrosin traced at 240 nm. (TIF)

S2 Fig Ambrosin chemical structure and important HMBC correlations. (TIF)

S1 Table Chemical shifts of 1H-NMR and 13C-NMR of ambrosin. (DOC)

## References

[1] Karunaweera N, Raju R, Gyengesi E, Münch G. Plant polyphenols as inhibitors of NF-κB induced cytokine production—a potential anti-inflammatory treatment for Alzheimer's disease? Frontiers in Molecular Neuroscience. 2015;8:24 10.3389/fnmol.2015.00024 PMC4468843. 26136655 PMC4468843

[2] Van Eldik LJ, Carrillo MC, Cole PE, Feuerbach D, Greenberg BD, Hendrix JA, et al The roles of inflammation and immune mechanisms in Alzheimer's disease. Alzheimer's & Dementia: Translational Research & Clinical Interventions. 2016;2(2):99–109. 10.1016/j.trci.2016.05.001 29067297 PMC5644267

[3] Shi Z-M, Han Y-W, Han X-H, Zhang K, Chang Y-N, Hu Z-M, et al Upstream regulators and downstream effectors of NF-κB in Alzheimer's disease. Journal of the neurological sciences. 2016;366:127–34. 10.1016/j.jns.2016.05.022 27288790

[4] Srinivasan M, Lahiri DK. Significance of NF-κB as a pivotal therapeutic target in the neurodegenerative pathologies of Alzheimer’s disease and multiple sclerosis. Expert opinion on therapeutic targets. 2015;19(4):471–87. 10.1517/14728222.2014.989834 PMC5873291. 25652642 PMC5873291

[5] Venigalla M, Sonego S, Gyengesi E, Sharman MJ, Munch G. Novel promising therapeutics against chronic neuroinflammation and neurodegeneration in Alzheimer's disease. Neurochem Int. 2016;95:63–74. Epub 2015/11/04. 10.1016/j.neuint.2015.10.011 . 26529297

[6] Goozee KG, Shah TM, Sohrabi HR, Rainey-Smith SR, Brown B, Verdile G, et al Examining the potential clinical value of curcumin in the prevention and diagnosis of Alzheimer’s disease. British Journal of Nutrition. 2015;115(3):449–65. Epub 12/14. 10.1017/S0007114515004687 26652155

[7] Serafini MM, Catanzaro M, Rosini M, Racchi M, Lanni C. Curcumin in Alzheimer’s disease: Can we think to new strategies and perspectives for this molecule? Pharmacological research. 2017;124:146–55. 10.1016/j.phrs.2017.08.004 28811228

[8] Seo EJ, Fischer N, Efferth T. Phytochemicals as inhibitors of NF-kappaB for treatment of Alzheimer's disease. Pharmacological research. 2018;129:262–73. Epub 2017/11/29. 10.1016/j.phrs.2017.11.030 . 29179999

[9] Abdelgaleil SAM, Badawy MEI, T. S, K. K. Antifungal and biochemical effects of pseudoguaianolide sesquiterpenes isolated from Ambrosia maritima L. African Journal of Microbiology Research. 2011;5:3385–92.

[10] Svensson D, Lozano M, Almanza GR, Nilsson B-O, Sterner O, Villagomez R. Sesquiterpene lactones from Ambrosia arborescens Mill. inhibit pro-inflammatory cytokine expression and modulate NF-κB signaling in human skin cells. Phytomedicine. 2018. 10.1016/j.phymed.2018.04.011. 30466970

[11] Sotillo WS, Villagomez R, Smiljanic S, Huang X, Malakpour A, Kempengren S, et al Anti-cancer stem cell activity of a sesquiterpene lactone isolated from Ambrosia arborescens and of a synthetic derivative. PloS one. 2017;12(9):e0184304 10.1371/journal.pone.0184304 28863191 PMC5581169

[12] Villagomez R, Collado J, Muñoz E, Almanza G, Sterner O. Natural and Semi-Synthetic Pseudoguaianolides as Inhibitors of NF-κB. Journal of Biomedical Science and Engineering. 2014;(7): 833–47.

[13] Cheng F, Li W, Zhou Y, Shen J, Wu Z, Liu G, et al admetSAR: A Comprehensive Source and Free Tool for Assessment of Chemical ADMET Properties. Journal of chemical information and modeling. 2012;52(11):3099–105. 10.1021/ci300367a 23092397

[14] Daina A, Michielin O, Zoete V. SwissADME: a free web tool to evaluate pharmacokinetics, drug-likeness and medicinal chemistry friendliness of small molecules. Scientific Reports. 2017;7:42717 10.1038/srep42717 https://www.nature.com/articles/srep42717#supplementary-information. 28256516 PMC5335600

[15] Herz W, Gage D, Kumar N. Damsinic acid and ambrosanolides from vegetative ambrosia hispida. Phytochemistry. 1981;20(7):1601–4. 10.1016/S0031-9422(00)98540-6.

[16] Qin L, Wu X, Block ML, Liu Y, Breese GR, Hong JS, et al Systemic LPS causes chronic neuroinflammation and progressive neurodegeneration. Glia. 2007;55(5):453–62. 10.1002/glia.20467 17203472 PMC2871685

[17] Ghantous A, Sinjab A, Herceg Z, Darwiche N. Parthenolide: from plant shoots to cancer roots. Drug discovery today. 2013;18(17–18):894–905. Epub 2013/05/22. 10.1016/j.drudis.2013.05.005 . 23688583

[18] Sanmukhani J, Anovadiya A, Tripathi CB. Evaluation of antidepressant like activity of curcumin and its combination with fluoxetine and imipramine: an acute and chronic study. Acta poloniae pharmaceutica. 2011;68(5):769–75. Epub 2011/09/21. . 21928724

[19] Bromley-Brits K, Deng Y, Song W. Morris water maze test for learning and memory deficits in Alzheimer's disease model mice. Journal of visualized experiments: JoVE. 2011;(53). Epub 2011/08/03. 10.3791/2920 21808223 PMC3347885

[20] Kenawy S, Hegazy R, Hassan A, El-Shenawy S, Gomaa N, Zaki H, et al Involvement of insulin resistance in D-galactose-induced age-related dementia in rats: Protective role of metformin and saxagliptin. PloS one. 2017;12(8):e0183565 10.1371/journal.pone.0183565 28832656 PMC5568415

[21] Mazumder AG, Sharma P, Patial V, Singh D. Crocin Attenuates Kindling Development and Associated Cognitive Impairments in Mice via Inhibiting Reactive Oxygen Species-Mediated NF-kappaB Activation. Basic & clinical pharmacology & toxicology. 2017;120(5):426–33. Epub 2016/11/02. 10.1111/bcpt.12694 . 27800651

[22] El-Marasy SA, El Awdan SA, Abd-Elsalam RM. Protective role of chrysin on thioacetamide-induced hepatic encephalopathy in rats. Chemico-Biological Interactions. 2019;299:111–9. 10.1016/j.cbi.2018.11.021 30500344

[23] Chui DH, Tanahashi H, Ozawa K, Ikeda S, Checler F, Ueda O, et al Transgenic mice with Alzheimer presenilin 1 mutations show accelerated neurodegeneration without amyloid plaque formation. Nature medicine. 1999;5(5):560–4. Epub 1999/05/06. 10.1038/8438 . 10229234

[24] Veber DF, Johnson SR, Cheng H-Y, Smith BR, Ward KW, Kopple KD. Molecular properties that influence the oral bioavailability of drug candidates. Journal of medicinal chemistry. 2002;45(12):2615–23. 12036371 10.1021/jm020017n

[25] van Breemen RB, Li Y. Caco-2 cell permeability assays to measure drug absorption. Expert opinion on drug metabolism & toxicology. 2005;1(2):175–85. Epub 2006/08/23. 10.1517/17425255.1.2.175 . 16922635

[26] Shih R-H, Wang C-Y, Yang C-M. NF-kappaB Signaling Pathways in Neurological Inflammation: A Mini Review. Frontiers in Molecular Neuroscience. 2015;8:77 10.3389/fnmol.2015.00077 PMC4683208. 26733801 PMC4683208

[27] Kaur U, Banerjee P, Bir A, Sinha M, Biswas A, Chakrabarti S. Reactive oxygen species, redox signaling and neuroinflammation in Alzheimer's disease: The NF-κB connection. Current topics in medicinal chemistry. 2015;15(5):446–57. 25620241 10.2174/1568026615666150114160543

[28] Hoogland ICM, Houbolt C, van Westerloo DJ, van Gool WA, van de Beek D. Systemic inflammation and microglial activation: systematic review of animal experiments. Journal of Neuroinflammation. 2015;12(1):114 10.1186/s12974-015-0332-6 26048578 PMC4470063

[29] Heneka MT, Carson MJ, El Khoury J, Landreth GE, Brosseron F, Feinstein DL, et al Neuroinflammation in Alzheimer's disease. The Lancet Neurology. 2015;14(4):388–405. 10.1016/S1474-4422(15)70016-5 25792098 PMC5909703

[30] D'Amelio M, Cavallucci V, Middei S, Marchetti C, Pacioni S, Ferri A, et al Caspase-3 triggers early synaptic dysfunction in a mouse model of Alzheimer's disease. Nature neuroscience. 2011;14(1):69 10.1038/nn.2709 21151119

[31] García-Piñeres AJ, Castro Vc, Mora G, Schmidt TJ, Strunck E, Pahl HL, et al Cysteine 38 in p65/NF-κB Plays a Crucial Role in DNA Binding Inhibition by Sesquiterpene Lactones. Journal of Biological Chemistry. 2001;276(43):39713–20. 10.1074/jbc.M101985200 11500489

[32] Wahlang B, Pawar YB, Bansal AK. Identification of permeability-related hurdles in oral delivery of curcumin using the Caco-2 cell model. European journal of pharmaceutics and biopharmaceutics: official journal of Arbeitsgemeinschaft fur Pharmazeutische Verfahrenstechnik eV. 2011;77(2):275–82. Epub 2010/12/15. 10.1016/j.ejpb.2010.12.006 . 21147222

[33] Nava Catorce M, Gevorkian G. LPS-induced murine neuroinflammation model: main features and suitability for pre-clinical assessment of nutraceuticals. Current neuropharmacology. 2016;14(2):155–64. 10.2174/1570159X14666151204122017 26639457 PMC4825946

[34] Jobin C, Bradham CA, Russo MP, Juma B, Narula AS, Brenner DA, et al Curcumin blocks cytokine-mediated NF-kappa B activation and proinflammatory gene expression by inhibiting inhibitory factor I-kappa B kinase activity. J Immunol. 1999;163(6):3474–83. Epub 1999/09/08. . 10477620

[35] Siedle B, García-Piñeres AJ, Murillo R, Schulte-Mönting J, Castro V, Rüngeler P, et al Quantitative structure− activity relationship of sesquiterpene lactones as inhibitors of the transcription Factor NF-κB. Journal of medicinal chemistry. 2004;47(24):6042–54. 10.1021/jm049937r 15537359

[36] Rüngeler P, Castro V, Mora G, Gören N, Vichnewski W, Pahl HL, et al Inhibition of transcription factor NF-κB by sesquiterpene lactones: a proposed molecular mechanism of action. Bioorganic & medicinal chemistry. 1999;7(11):2343–52. 10.1016/S0968-0896(99)00195-9. 10632044

[37] Hehner SP, Heinrich M, Bork PM, Vogt M, Ratter F, Lehmann V, et al Sesquiterpene lactones specifically inhibit activation of NF-κB by preventing the degradation of IκB-α and IκB-β. Journal of Biological Chemistry. 1998;273(3):1288–97. 10.1074/jbc.273.3.1288 9430659

[38] Youl Cho J. Sesquiterpene Lactones as a Potent Class of NF-kB Activation Inhibitors. Current Enzyme Inhibition. 2006;2(4):329–41. 10.2174/157340806778699299

[39] Cavin C, Delannoy M, Malnoe A, Debefve E, Touche A, Courtois D, et al Inhibition of the expression and activity of cyclooxygenase-2 by chicory extract. Biochem Biophys Res Commun. 2005;327(3):742–9. Epub 2005/01/15. 10.1016/j.bbrc.2004.12.061 . 15649409

[40] Choodej S, Pudhom K, Mitsunaga T. Inhibition of TNF-alpha-Induced Inflammation by Sesquiterpene Lactones from Saussurea lappa and Semi-Synthetic Analogues. Planta medica. 2018;84(5):329–35. Epub 2017/09/30. 10.1055/s-0043-120115 . 28962049

[41] Kitazawa M, Cheng D, Tsukamoto M, Koike M, Wes PD, Vasilevko V, et al Blocking Interleukin-1 Signaling Rescues Cognition, Attenuates Tau Pathology, and Restores Neuronal β-Catenin Pathway Function in an Alzheimer's Disease Model. Journal of immunology (Baltimore, Md: 1950). 2011;187(12):6539–49. 10.4049/jimmunol.1100620 PMC4072218. 22095718 PMC4072218

[42] Koch E, Klaas CA, Rungeler P, Castro V, Mora G, Vichnewski W, et al Inhibition of inflammatory cytokine production and lymphocyte proliferation by structurally different sesquiterpene lactones correlates with their effect on activation of NF-kappaB. Biochem Pharmacol. 2001;62(6):795–801. Epub 2001/09/12. 10.1016/s0006-2952(01)00714-6 . 11551526

[43] Chen CH, Zhou W, Liu S, Deng Y, Cai F, Tone M, et al Increased NF-kappaB signalling up-regulates BACE1 expression and its therapeutic potential in Alzheimer's disease. The international journal of neuropsychopharmacology. 2012;15(1):77–90. Epub 2011/02/19. 10.1017/S1461145711000149 . 21329555

[44] Minett T, Classey J, Matthews FE, Fahrenhold M, Taga M, Brayne C, et al Microglial immunophenotype in dementia with Alzheimer's pathology. J Neuroinflammation. 2016;13(1):135 Epub 2016/06/04. 10.1186/s12974-016-0601-z 27256292 PMC4890505

[45] Li X, Long J, He T, Belshaw R, Scott J. Integrated genomic approaches identify major pathways and upstream regulators in late onset Alzheimer’s disease. Scientific Reports. 2015;5:12393 10.1038/srep12393 https://www.nature.com/articles/srep12393#supplementary-information. 26202100 PMC4511863

[46] Saeed M, Jacob S, Sandjo LP, Sugimoto Y, Khalid HE, Opatz T, et al Cytotoxicity of the Sesquiterpene Lactones Neoambrosin and Damsin from Ambrosia maritima Against Multidrug-Resistant Cancer Cells. Frontiers in pharmacology. 2015;6:267 Epub 2015/12/01. 10.3389/fphar.2015.00267 26617519 PMC4637410

[47] Amoah SKS, Dalla Vecchia MT, Pedrini B, Carnhelutti GL, Gonçalves AE, dos Santos DA, et al Inhibitory effect of sesquiterpene lactones and the sesquiterpene alcohol aromadendrane-4β,10α-diol on memory impairment in a mouse model of Alzheimer. European Journal of Pharmacology. 2015;769:195–202. 10.1016/j.ejphar.2015.11.018 26593432

[48] Hegazy M-EF, Ibrahim AY, Mohamed TA, Shahat AA, El Halawany AM, Abdel-Azim NS, et al Sesquiterpene Lactones from Cynara cornigera: Acetyl Cholinesterase Inhibition and In Silico Ligand Docking. Planta medica. 2016;82(1–2):138–46. 10.1055/s-0035-1558088 . 26441064

[49] Gupta SC, Patchva S, Aggarwal BB. Therapeutic roles of curcumin: lessons learned from clinical trials. The AAPS journal. 2012;15(1):195–218. 10.1208/s12248-012-9432-8 . 23143785 PMC3535097

