# Supplementary figures and images for: Ambrosin, a potent NF-κβ inhibitor, ameliorates lipopolysaccharide induced memory impairment, comparison to curcumin
Source: PLoS One. 2019 Jul 5;14(7):e0219378. doi: 10.1371/journal.pone.0219378 (PMC6611615; doi:10.1371/journal.pone.0219378)

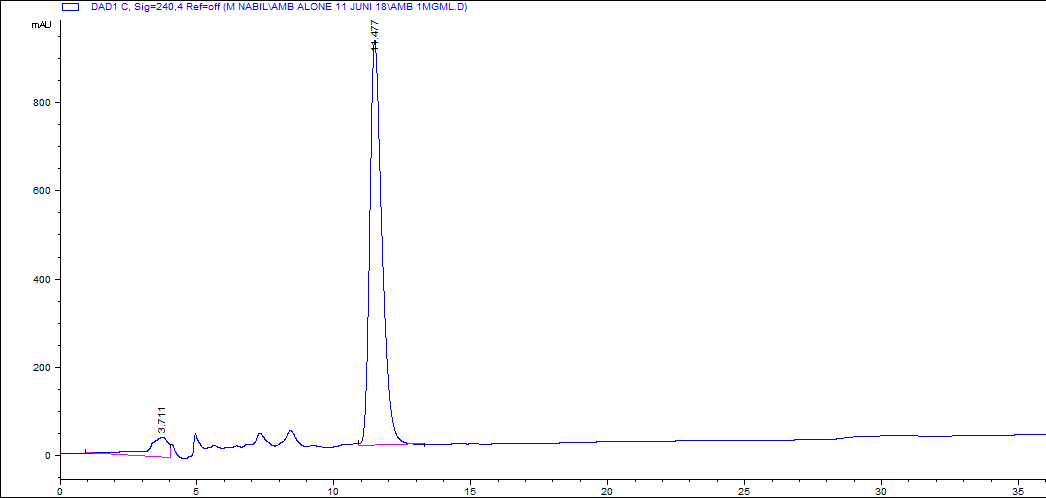

Supplement: S1 Fig — (TIF) [file pone.0219378.s001.tif]

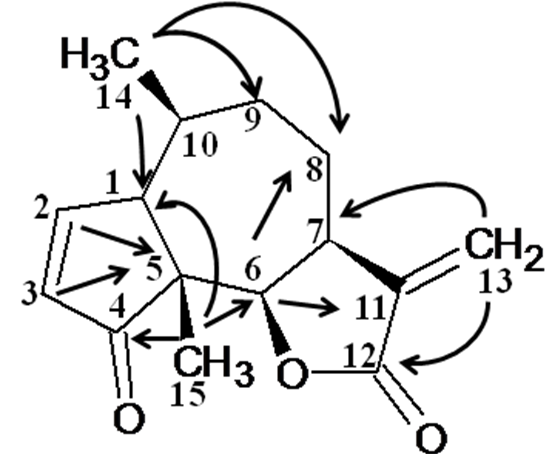

Supplement: S2 Fig — (TIF) [file pone.0219378.s002.tif]
