# Supplementary material for: Ambrosin, a potent NF-κβ inhibitor, ameliorates lipopolysaccharide induced memory impairment, comparison to curcumin
Source: PLoS One. 2019 Jul 5;14(7):e0219378. doi: 10.1371/journal.pone.0219378 (PMC6611615; doi:10.1371/journal.pone.0219378)
Supplement: S1 Table — (DOC) [file pone.0219378.s003.doc]

**For submission:** *Plos one*

**Title: Ambrosin, a potent NF-κβ inhibitor, ameliorates lipopolysaccharide induced memory impairment, comparison to curcumin.**

**Author Names**

**Mohammed N. A. Khalil^1,*^, Mouchira A. Choucry^1, #^, Amira S. El Senousy^1^, Azza Hassan^2^, Salma A. El-Marasy^3^, Sally A. El Awdan^3^, Farghaly A. Omar^4^**

**^1^ Pharmacognosy Department, Faculty of Pharmacy, Cairo University,**

**Cairo, Egypt.**

**^2^ Pathology Department, Faculty of Veterinary Medicine, Cairo University,**

**Giza Square, Giza, Egypt.**

**^3^ Pharmacology Department, National Research Centre, Giza, Egypt.**

**^4^ Pharmaceutical Chemistry Department, Faculty of Pharmacy, Assiut University, Assiut, Egypt.**

**^#^Current address: Pharmacognosy Department, Faculty of Pharmacy, Heliopolis University, Cairo, Egypt.**

**^*^ Corresponding author**

**E-mail: mohamed.nabil@pharma.cu.edu.eg**

**Short title: Pharmacological activities of ambrosin, drug repurposing as a remedy for Alzheimer**

**S1 Table. Chemical shifts of ^1^H-NMR and ^13^C-NMR of ambrosin**

| **Number** | **^1^H-NMR (400 MHz, CDCl_3_)** | **^13^C-NMR (100MHz, CDCl_3_)** |
| --- | --- | --- |
| 1 | 2.99 (1H, m, H-1) | 47.78 |
| 2 | 7.49 (1H, dd, J = 1.9, 5.88 Hz, H-2) | 163.29 |
| 3 | 6.16 (1H, dd, J = 2.9, 5.88 Hz, H-3) | 131.19 |
| 4 | -------------- | 210 |
| 5 | -------------- | 56.2 |
| 6 | 4.67 (1H, d, J = 8.57 Hz, H-6) | 80.01 |
| 7 | 3.45 (1H, m, H-7), | 44.52 |
| 8 | 2.45 (1H, m, H-8a)  1.9 (1H, m, H-8b) | 24.77 |
| 9 | 1.88 (1H, m, H-9a)  1.74 (1H, m, H-9b), | 29.67 |
| 10 | 2.44 (1H, m, H-10) | 33.78 |
| 11 | -------------- | 138.11 |
| 12 | -------------- | 170.43 |
| 13 | 6.31 (1H, d, J = 3.4 Hz, H-13a)  5.51 (1H, d, J = 3.16 Hz, H-13b) | 119.9 |
| 14 | 1.055 (3H, d, J = 7.4 Hz, H-14) | 17.4 |
| 15 | 1.26 (3H, s, H-15), | 16.75 |
